# Iodine Close Packing in Hybrid Halide Bismuth(III) and Antimony(III) Semiconductors: (NH_3_(CH_2_)_7_NH_3_)_2_Bi_2_I_10_ and (NH_3_(CH_2_)_7_NH_3_)_2_Sb_2_I_10_

**DOI:** 10.1021/acs.inorgchem.6c01909

**Published:** 2026-06-06

**Authors:** Shelby R. Lane, Dominic Cudjoe Asebiah, Alexander G. Squires, Autumn N. Peters, Luke T. MacHale, Lauren Borgia, Mirella K. Villani, David O. Scanlon, Obadiah G. Reid, James R. Neilson

**Affiliations:** † Department of Chemistry, 3447 Colorado State University, Fort Collins, Colorado 80523, United States; ‡ School of Materials Science & Engineering, 3447 Colorado State University, Fort Collins, Colorado 80523, United States; ¶ School of Chemistry, University of Birmingham, Edgbaston, Birmingham B15 2TT, U.K.; § Renewable and Sustainable Energy Institute, University of Colorado Boulder, Boulder, Colorado 80303, United States; ∥ Chemistry & Nanoscience Center, National Laboratory of the Rockies, Golden, Colorado 80401, United States

## Abstract

Simple features in complex hybrid inorganic–organic crystalline materials provide opportunities for targeted discovery of materials with desired optoelectronic properties. In this study, we report the structure and optoelectronic properties of isostructural (NH_3_(CH_2_)_7_NH_3_)_2_Bi_2_I_10_ and (NH_3_(CH_2_)_7_NH_3_)_2_Sb_2_I_10_. The crystal structures are characterized by corner-connected metal-iodide octahedral chains that form a cubic close-packed iodine inorganic framework. Variable temperature UV–visible diffuse reflectance spectroscopy reveals stark contrasts in the onset of absorption and color changes between the [MX_6_]^3–^ based structures, due to differences in the interaction of the (NH_3_(CH_2_)_7_NH_3_)^2+^ organic ammonium cation and the iodine packing of the inorganic framework. Density functional theory (DFT) calculations reveal flat bands reflective of the pseudo-1D crystal structure. Dark microwave conductivity (DMC) and time-resolved microwave conductivity (TRMC) reveal an excitonic character with long carrier lifetimes, consistent with the electronically confined octahedral chains. Comparison of the structural features with those of other diammonium-containing crystals reveals that diammoniumheptane can substitute into structures, displacing inorganic octahedra while retaining a close-packed anion framework. This provides a means for targeting new hybrid materials in which “vacancy-ordering” provides a crystal chemical approach for targeting desirable optoelectronic properties.

## Graphical abstract

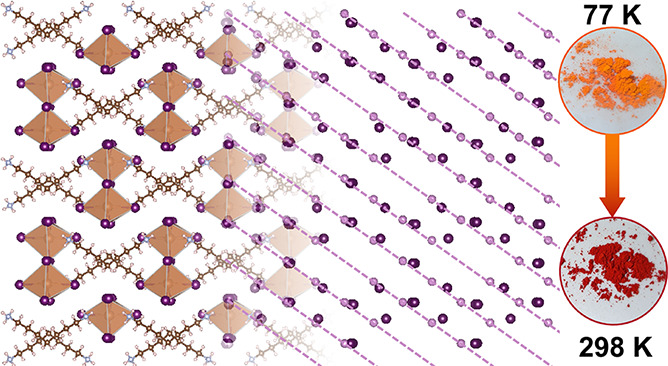

## Introduction

Hybrid halide perovskites, of the ABX_3_ formula (A = methylammonium, formamidinium, etc., B = Pb^2+^, Sn^2+^, X = Cl^–^, Br^–^, I^–^) have shown applications in many areas of technology such as neuromorphic computing,^1^ thermoelectrics,^2^ radiation detectors,^3^ and photovoltaics.^4–7^ Pb­(II)-based halide perovskites have exhibited excellent optoelectronic properties due to long carrier lifetimes,^8^ long-carrier diffusion lengths,^9–11^ high absorption coefficients,^12,13^ low exciton binding energy,^14–16^ and small effective carrier masses,^17^ with origins in strong antibonding coupling between the valence and conduction band^18^ and a stereochemically active lone-pair.^12,19–22^ However, longstanding concerns regarding lead toxicity are a limiting factor in production and device implementation. Sn­(II)-based halide perovskites have proven to be a viable alternative but suffer from oxidation from Sn­(II) to Sn­(IV), which yields metallic-like behavior and devices susceptible to thermal and oxidative degradation.^23–25^ Group V metals like Bi­(III) and Sb­(III) are ideal alternative metal ions due to ionic radii similar to that of Sn^2+^ and Pb^2+^,^26^ the presence of *n*s^2^-derived electron configurations compatible with stereochemically active lone pairs, decreased toxicity, and increased stability.^27,28^

In these materials, the inorganic framework, composed of corner-linked octahedra, dominates the electronic structure, where the metal-derived *s*-orbital and the anion *p*-orbitals mix to comprise the valence band.^8^ However, the organic cation can indirectly influence electronic properties through modification of the crystal structure. Tailoring the size, chirality, and bonding characteristics of the organic cation has been used to promote highly diverse structural motifs^29–33^ and electronic properties.^34,35^ For example, the Dion-Jacobson and Ruddlesden–Popper perovskite-derived structures are often synthetically targeted with a large organic cation that promotes the formation of 2D sheets of octahedra, rather than the 3D corner-connected aristotype.^36,37^ The spatial confinement of these 2D materials leads to unique electronic properties and increased thermodynamic stability,^38,39^ warranting further examination of other reduced dimensionality structural motifs.

One such class of materials comprises vacancy-ordered perovskite-derived structures. Vacancy-ordered 0D double perovskites, of the formula A_2_B□X_6_ (where □ is a vacancy), show high tunability of electronic properties strongly dependent on the inorganic framework.^40–43^ However, synthetic routes targeting vacancy-ordered structures are less understood, especially those of higher dimensionality that are ideal for semiconducting applications. One interesting 3D vacancy-ordered perovskite is that of (NH_3_(CH_2_)_7_NH_3_)_2_Sn_3_I_10_, where layers of [SnI_6_]^4–^ are bridged with [SnI_5_]^3–^ square pyramids,^44^ likely promoted by the conformation of the large diammoniumheptane (NH_3_(CH_2_)_7_NH_3_)^2+^ organic cation to space the octahedral layers ideally. This has been seen in some bismuth-containing compounds, such as (NH_3_(CH_2_)_7_NH_3_)_2_Bi_2_Cl_10_ ^45^ and (NH_3_(CH_2_)_7_NH_3_)_2_Bi_2_I_10_.^46^ The impact of dominant B-site and X-site vacancies^47,48^ can be isolated and studied in this context, which is necessary for understanding targeted synthesis of novel and highly tunable vacancy-ordered perovskite semiconducting materials. There, the length of the diammoniumheptane cation appears consistent with a I–Sn–I segment, suggesting that the cation may be used to template other crystal structures with closed-packed anions.

Herein, we describe the synthesis and structural characterization of two Bi­(III) and Sb­(III) perovskite-derived, vacancy-ordered semiconducting materials, (NH_3_(CH_2_)_7_NH_3_)_2_Bi_2_I_10_ and (NH_3_(CH_2_)_7_NH_3_)_2_Sb_2_I_10_, that have reduced electronic dimensionality. The crystal structure and anion close packing are characterized by single crystal X-ray diffraction, high-resolution synchrotron powder X-ray diffraction, and X-ray pair distribution function analysis. Analysis of the iodine cubic close packing (*ABC* stacking) shows electronic properties of (NH_3_(CH_2_)_7_NH_3_)_2_Bi_2_I_10_ to be dominated by the anion-packed inorganic framework, and those of (NH_3_(CH_2_)_7_NH_3_)_2_Sb_2_I_10_ to be dominated by the greater flexibility of the organic cation within the more distorted inorganic framework. Optical properties were examined using powdered specimens and compared to density functional theory calculations of the electronic structures. In addition, dark and time-resolved microwave conductivity measurements were performed on powdered samples to evaluate electrical conductivity, carrier mobility, carrier density, and dielectric permittivity. Comparison of crystal chemical features with other diammonium-containing crystals reveals shared trends for replacing octahedral with diammoniumheptane to yield “vacancy-ordered” perovskite derivatives.

## Methods

### Starting Materials

Tin (granular, 20 mesh, 99%, J.T. Baker Chemical Co.), antimony (powdered, >99.5%, Fisher Scientific), iodine (>99.8%, Sigma-Aldrich), 1,7-diaminoheptane (98%, Sigma-Aldrich), bismuth­(III) iodide (99%, Sigma-Aldrich), and hydroiodic acid (57% in H_2_O, stabilized with H_3_PO_2_, Sigma-Aldrich) were purchased from the specified commercial suppliers and used as received without further purification.

### Synthesis of SbI_3_

Solid iodine (3.8202 g, 15.05 mmol) was ground into a homogeneous fine powder using an agate mortar and pestle and mixed with powdered antimony (1.2318 g, 10.17 mmol) in a 3:2 ratio and added to a 10 × 12 mm (ID:OD) quartz tube. The tube was briefly evacuated (<5 s to negate loss of iodine via sublimation) and flame-sealed. The ampule was heated in a muffle furnace to 200 °C over 10 min, held at the temperature for 60 h, and cooled in the furnace to room temperature. The resulting dark red powder was ground and confirmed pure by PXRD with preferred orientation along the {*h*00} planes.

### Synthesis of (NH_3_(CH_2_)_7_NH_3_)_2_M_2_I_10_

Stoichiometric amounts of finely ground bismuth­(III) iodide (0.5882, 1.00 mmol) or antimony­(III) iodide (0.5022 g, 1.00 mmol) and 1,7-diaminoheptane (0.1309 g, 1.00 mmol) were weighed and combined in an oven-dried round-bottom flask under an inert atmosphere. Cold (0 °C, ice bath), concentrated hydroiodic acid (HI, 57 wt %, 6.5 mL) was carefully introduced via syringe while nitrogen gas was continuously passed through the system to prevent oxygen exposure. The reaction flask was lowered into a temperature-controlled oil bath and heated to 135 °C while stirring at 300 rpm until complete dissolution, and then refluxed for 2 h, producing a cloudy, bright red solution. For the bulk powder product, the reaction flask was quenched in an ice bath, washed with toluene, and dried under vacuum. To promote crystal growth, a slow cool of the reaction flask to a predetermined temperature allowed formation of crystals over 1–28 d. (NH_3_(CH_2_)_7_NH_3_)_2_Bi_2_I_10_ was dark red and (NH_3_(CH_2_)_7_NH_3_)_2_Sb_2_I_10_ was light red.

### UV–visible Diffuse Reflectance Spectroscopy

Diffuse reflectance optical spectroscopy measurements were performed using an Ocean Optics tungsten-halogen light source (Mikropack HL-2000) and an Ocean Insight Flame miniature spectrometer (Flame-S-VIS-NIR) equipped with an integrating sphere. A fiber-optic cable directed light onto the flat base of a borosilicate glass vial containing loose, finely ground powder of the reaction products for measurements. A similarly mounted vial filled with BaSO_4_ served as a white reflectance standard. To maintain an inert environment, the sample vials were sealed with electrical tape inside an argon-filled glovebox before being removed for measurement. For measurements at 100 K, sample vials were suspended in a dewar of liquid nitrogen until equilibrated (≈one min). The vial was wiped to remove any condensation and quickly placed on the integrating sphere, where the vial was left until warm, evidenced by a return to dark red color and comparison to a room temperature sample reference. Measurements were taken every second for 30 s while warming to fully capture transitions in reflectance. The Kubelka–Munk transformation was applied using the equation (*k*/*s*) = ((1 – *R*_*∞*_)^2^)/(2*R*_*∞*_), where *k* is the absorption coefficient, *s* is the scattering coefficient, and *R*_*∞*_ represents the diffuse reflectance. The optical band gap was estimated by determining the tangent line at the maximum slope point on the absorption edge and extrapolating to *k*/*s* = 0.

### X-ray Diffraction and Scattering

#### Single-Crystal X-ray Diffraction (SCXRD)

Single-crystal X-ray diffraction (SCXRD) data were collected at Colorado State University using a Bruker D8 Advance Quest diffractometer equipped with an Incoatec (I μS) microfocus X-ray source and dual microfocus systems. Crystals ≈50–100 μm in size were selected under paratone oil and mounted on a B3-R MicroRT goniometer using 100 μm micromounts. An Oxford Cryostream system was used to cool the samples gradually to 100 K, providing thermal stability throughout data acquisition. Additional measurements taken at 300 and 150 K indicated that twinning was inherent to the crystal growth process, rather than induced by thermal changes. Data were collected using monochromatic Mo Kα radiation (λ = 0.71073 Å), and processed with the Bruker APEX4 ^49^ software package. Two twin domains were identified and subsequently detwinned. Nonmerohedral twinned domains are related by a 180° rotation about the – c* axis. The frames were scaled, merged, and absorption-corrected using TWINABS.^50^ The crystal structure was initially solved in Olex2.^51^ Structural refinement was performed using SHELXT^52^ based on reflections corresponding to the primary twin domain, followed by a refinement of the two twin domains using SHELXL with detwinned reflection data in HKLF5 format within Olex2. The solved single-crystal structure was used as the initial model for the Rietveld refinement of in-house and synchrotron powder X-ray diffraction (PXRD) data.

#### Powder X-ray Diffraction (PXRD)

Laboratory powder X-ray diffraction (PXRD) data were acquired using a Bruker D8 Discover diffractometer with a Cu Kα radiation source and a Lynxeye XE-T position-sensitive detector to confirm phase purity. Samples were finely ground with an agate mortar and pestle and mounted on a “zero diffraction” silicon wafer for measurement. The diffraction data were subsequently analyzed using the Rietveld refinement method, modeled against the obtained SCXRD structure, implemented in TOPAS v6. (NH_3_(CH_2_)_7_NH_3_)_2_Sb_2_I_10_ was determined to have preferred orientation along the {*h*00} planes, which was accounted for using a March-Dollase model. Air stability of both samples was confirmed via no change in PXRD patterns for upward of six months.

### Total Scattering and Pair Distribution Function Analysis

Total scattering data for (NH_3_(CH_2_)_7_NH_3_)_2_Bi_2_I_10_ and (NH_3_(CH_2_)_7_NH_3_)_2_Sb_2_I_10_ were collected at room temperature on beamline 28-ID-1 and 28-ID-2, respectively, at the National Synchrotron Light Source II (NSLS-II) using an X-ray wavelength of 0.1665 Å­(Bi) or 0.181 Å­(Sb). Before data acquisition, powder samples were flame-sealed under vacuum in 1.2 × 1.4 mm (ID:OD) borosilicate (1.0 × 1.1 mm polyimide) capillaries to ensure sample integrity. Diffraction measurements were conducted with a sample-to-detector distance of approximately 217.7965 mm (Bi) and 216.3279 mm (Sb), enabling a usable *Q*_max_ = 26 Å^–1^ (Bi) or 25 Å^–1^ (Sb). Data was collected using a wide-angle PerkinElmer image plate detector (2048 × 2048 array, 200 μm pixel pitch). Each sample underwent two X-ray exposures, with a total exposure time of 60 s per sample, consisting of a 0.5 s (Bi) 0.2 s (Sb) detector exposure followed by a 60 s sleep period between exposures. To enhance data quality, the two images were averaged. The detector’s position and alignment were calibrated using CeO_2_. The collected diffraction data were normalized into *S*(*Q*) and subsequently transformed into the pair distribution function (PDF) using the equation $G(r)=\frac{2}{\pi }\int_{Q_{\min}}^{Q_{\max}}Q[S(Q)-1]\sin(Qr)dQ$ . This transformation was performed using PDFgetX3,^53^ where the model refinements to the experimental PDFs were carried out with PDFgui.^54^ Additionally, instrumental factors were determined using polycrystalline Ni powder measurements to ensure accuracy in subsequent analyses, with the following parameters: *Q*_max_ = 26 Å^–1^ (Bi) or 25 Å^–1^ (Sb), *Q*_broad_ = 0.02011 Å^–1^ (Bi) or 0.02011 Å^–1^ (Sb), and *Q*_damp_ = 0.03506 Å^–1^, as defined by PDFgui.^54^

### Iodine Close Packing Analysis

The ISOTROPY Software Suite tool ISODISTORT^55,56^ was used to determine ideal iodine *ABC* close packing referenced to the (NH_3_(CH_2_)_7_NH_3_)_2_M_2_I_10_ (M = Bi^3+^, Sb^3+^) structural setting. An iodine-only *Fm*3̅*m* unit cell was constructed such that the lattice parameters (*a* = *b* = *c*) produced I–I distances equal to those of the average experimental structure values. For example, (NH_3_(CH_2_)_7_NH_3_)_2_Bi_2_I_10_, with an average I–I distance = 4.388(5) Å between close-packed layers, used a *Fm*3̅*m* lattice parameter of *a* = 6.206(3) Å to produce an I–I distance = 4.388(5) Å between close-packed layers. (NH_3_(CH_2_)_7_NH_3_)_2_Sb_2_I_10_, with an average I–I distance = 4.340(7) Å between close packed layers, used a *Fm*3̅*m* lattice parameter of *a* = 6.138(7) Å to produce an I–I distance = 4.340(7) Å between close-packed layers. The *Fm*3̅*m* structure was designated as the parent structure in ISODISTORT, where strain, displacive, and occupancy distortions were considered as decomposition modes onto the (“distorted”) experimental structures of (NH_3_(CH_2_)_7_NH_3_)_2_M_2_I_10_ (M = Bi^3+^, Sb^3+^), with all noniodine atoms removed. The iodine close packing was calculated as a symmetry allowed transformation based on the ideal *Fm*3̅*m* iodine close packing (<10% tolerance) to produce an ideally *ABC* iodine close-packed supercell in the same space group, *C*2/*c*, No. 15, with similar lattice parameters to the experimental structure using the basis transformation:

$$\left(\begin{array}{ccc} 2 & 0 & 0\\ 0 & 2 & -2\\ 0 & 1 & -1 \end{array}\right)$$ 1

The Bilbao crystallographic server tool COMPSTRU^57^ was used to quantify the difference in atomic positions and lattice deformation strain between iodine-only experimental and ideal structures. Atomic positions (tolerance = 3 Å) and lattice parameters (tolerance = 1 Å­(*a*, *b*, *c*) or 2° (β)) were compared between ideal and real structures, measured as a function of lattice deformation strain (*s*), maximum distance between matching ideal and real iodine positions (*d*_max_), average distance between the matching ideal and iodine positions (*d*_avg_), and the measure of similarity between ideal and real structures (δ).

### Density Functional Theory Calculations

Density functional theory calculations were carried out using the projector augmented wave approach within the Vienna ab initio Simulation Package (VASP).^58–61^ Structural relaxations were initiated from the experimentally determined crystal structure using the HSE06 hybrid functional,^62,63^ including Grimme’s D3 dispersion correction.^64^ Electronic band structures and densities of states were subsequently determined using HSE06 with spin–orbit coupling to account for the presence of heavy elements. A plane-wave energy cutoff of 450 eV and a 2 × 2 × 2 Monkhorst–Pack k-point mesh for the primitive cell were employed, with both parameters converged to within 1 meV per atom in total energy. Band structures and density of states were plotted using Sumo.^65^

Effective polar optical phonon (POP) frequencies were computed using density functional perturbation theory (DFPT) with the PBEsol functional. To account for the contribution of each phonon mode, an “effective phonon frequency” was employed, in which modes are weighted according to their induced dipole moment;^66^ high-frequency intramolecular modes were excluded from this treatment.^67^ Elastic constants were obtained through finite difference calculations, also employing the PBEsol functional. High-frequency dielectric constants were determined using hybrid DFT optical absorption calculations incorporating spin–orbit coupling. For anisotropic quantities, scalar values were obtained by taking the trace average of the dielectric tensors and the harmonic mean of the direction-dependent effective masses.

From these DFT-derived parameters, polaron properties were calculated within Feynman’s variational path-integral framework as implemented in PolaronMobility.jl,^67,68^ using the Fröhlich coupling constant α computed from the high-frequency and static dielectric constants, the effective phonon frequency, and the band effective mass. Polaron mobilities were evaluated at 300 K using Wannier–Mott exciton properties were estimated within effective mass theory, where the exciton Bohr diameter *a*_0_ and binding energy *E*_b_ are given by

$$a_{0}=\frac{2\hbar^{2}\varepsilon_{\infty }}{m^{*}e^{2}},\;E_{b}=\frac{2\hbar^{2}}{m^{*}a_{0}^{2}}$$ 2

where ε_*∞*_ is the high-frequency dielectric constant and m* is the reduced carrier mass.^69^

### Time-Resolved Microwave Conductivity (TRMC) Measurements

Time-resolved and dark microwave conductivity (TRMC) measurements were conducted following established methods.^70–73^ In brief, the samples were enclosed in either quartz EPR tubes (3 mm OD) or borosilicate capillaries, and electromagnetic simulations of the microwave cavity response function were used to connect both equilibrium and time-resolved changes in the cavity characteristics with the complex conductivity (permittivity) of the samples. To first order, the resonance frequency shift provides information on the dielectric constant, while variations in resonance depth and width were used to assess changes in real conductivity.

Dark conductivity was measured by evaluating power reflection coefficients across microwave frequencies near resonance using previously described instrumentation.^73^ Measurements were conducted in four conditions: a short-terminated waveguide, an empty cavity, a cavity containing a blank borosilicate capillary (ID:OD, 1.2:1.4 mm), and a cavity with the sample. The relative change in cavity characteristics between the empty capillary and that filled with the sample is analyzed as noted above to obtain the complex conductivity (permittivity). The measured values were corrected to account for sample filling, *f*, of the tubes using a simple linear effective medium approximation: the measured properties of the powder arise from a volume weighted average of air (ϵ = 1 – 0*i*) and the semiconductor. Each sample was packed into 4 borosilicate capillaries, and an average filling fraction, *f*, was determined to be ≈39% and ≈34% for (NH_3_(CH_2_)_7_NH_3_)_2_Bi_2_I_10_ and (NH_3_(CH_2_)_7_NH_3_)_2_Sb_2_I_10_, respectively, using the measured powder density (ρ_powder_) and the theoretical crystal density (ρ_crystal_): *f* = (ρ_powder_)/(ρ_crystal_). The real part of the sample permittivity is then calculated as $\epsilon_{r}^{\prime }=\frac{\epsilon_{\mathrm{powder}}^{\prime }-(1-f)}{f}$ and the imaginary part as $\epsilon_{i}^{\prime \prime }=\frac{\epsilon_{\mathrm{powder}}^{\prime \prime }}{f}$ .

The sensitivity factor for the real conductivity in TRMC measurements is obtained by fitting the cavity characteristics with electromagnetic simulations and taking the partial derivative of the microwave power reflectance on-resonance with respect to the sample conductivity.^70,72,74^ Monitoring changes in resonance depth alone was sufficient for semiconductors where real conductivity predominantly determined transient responses, as presented in Figures S1 and S2.

Photoconductivity data were used to compute real yield-mobility products, assuming powders absorbed all incident light within their projected cross-section. Transients were analyzed using a multiexponential function convolved with an instrument response function, with TRMC transients and corresponding fits recorded for all samples. Photoexcitation was performed using a 355 nm-pumped optical parametric oscillator (OPO) laser system (Continuum Surelite/Panther), with excitation wavelengths of 355 and 600 nm, as depicted in Figure 7. Dark conductivity and dielectric constant measurement uncertainties primarily result from film shape, substrate alignment, and cavity assembly variations.

### Diammonium Cation Length Analysis

N–N distances in 731 diammonium cations were calculated from 525 crystal structures in the Cambridge Structural Database. The shortest bonded path between terminal nitrogen atoms was determined using Dijkstra’s algorithm, and the end-to-end distance was calculated by summing the MIC displacement vectors along this path.^75–77^ This method accurately accounts for periodic boundary conditions and resolves molecules spanning unit cell boundaries. A detailed description is provided in the Supporting Information.

## Results and Discussion

### Structural Characterization

The synthesis of the title compounds is completed via a one-pot, air-free reaction of MI_3_ (M = Bi^3+^, Sb^3+^) and 1,7-diaminoheptane in a stirred solution of refluxing HI. Dark red bulk powder is produced upon quenching the solution in an ice bath, or twinned needle-like single crystals (≈1–2 mm on the largest side) are produced upon slow cooling. Structural characterization was performed via single-crystal X-ray diffraction (SCXRD), laboratory powder X-ray diffraction (PXRD), and high-resolution synchrotron PXRD.

(NH_3_(CH_2_)_7_NH_3_)_2_M_2_I_10_ crystallizes in the monoclinic space group *C*2/*c* (experimental crystallographic data are presented in Table 1) as corrugated 1D corner-connected chains of [MI_6_]^3–^ octahedra separated with (NH_3_(CH_2_)_7_NH_3_)^2+^ diammonium cations, as shown in Figure 1. The compounds are isostructural, where the [MI_6_]^3–^ octahedral chains arrange to produce a close-packed iodine lattice along the *c*-axis, emphasized in Figure 1a,b. The larger Bi­(III) cation is accommodated by an expansion in unit cell parameters and [MI_6_]^3–^ octahedra relative to Sb­(III). The corner-connected octahedra are staggered with an interchain I–I spacing of 8.2310(1) Å and 8.3595(1) Å for (NH_3_(CH_2_)_7_NH_3_)_2_Sb_2_I_10_ and (NH_3_(CH_2_)_7_NH_3_)_2_Bi_2_I_10_, respectively, interspersed with alternately oriented (NH_3_(CH_2_)_7_NH_3_)^2+^ cations, where the – NH_3_^+^ group is hydrogen-bonded to the iodine framework. For (NH_3_(CH_2_)_7_NH_3_)_2_Sb_2_I_10_, cooling to 100 K with liquid nitrogen was required to fully elucidate (NH_3_(CH_2_)_7_NH_3_)^2+^ atomic positions, suggestive of higher motion and flexibility of the organic cation.

**Figure 1:**
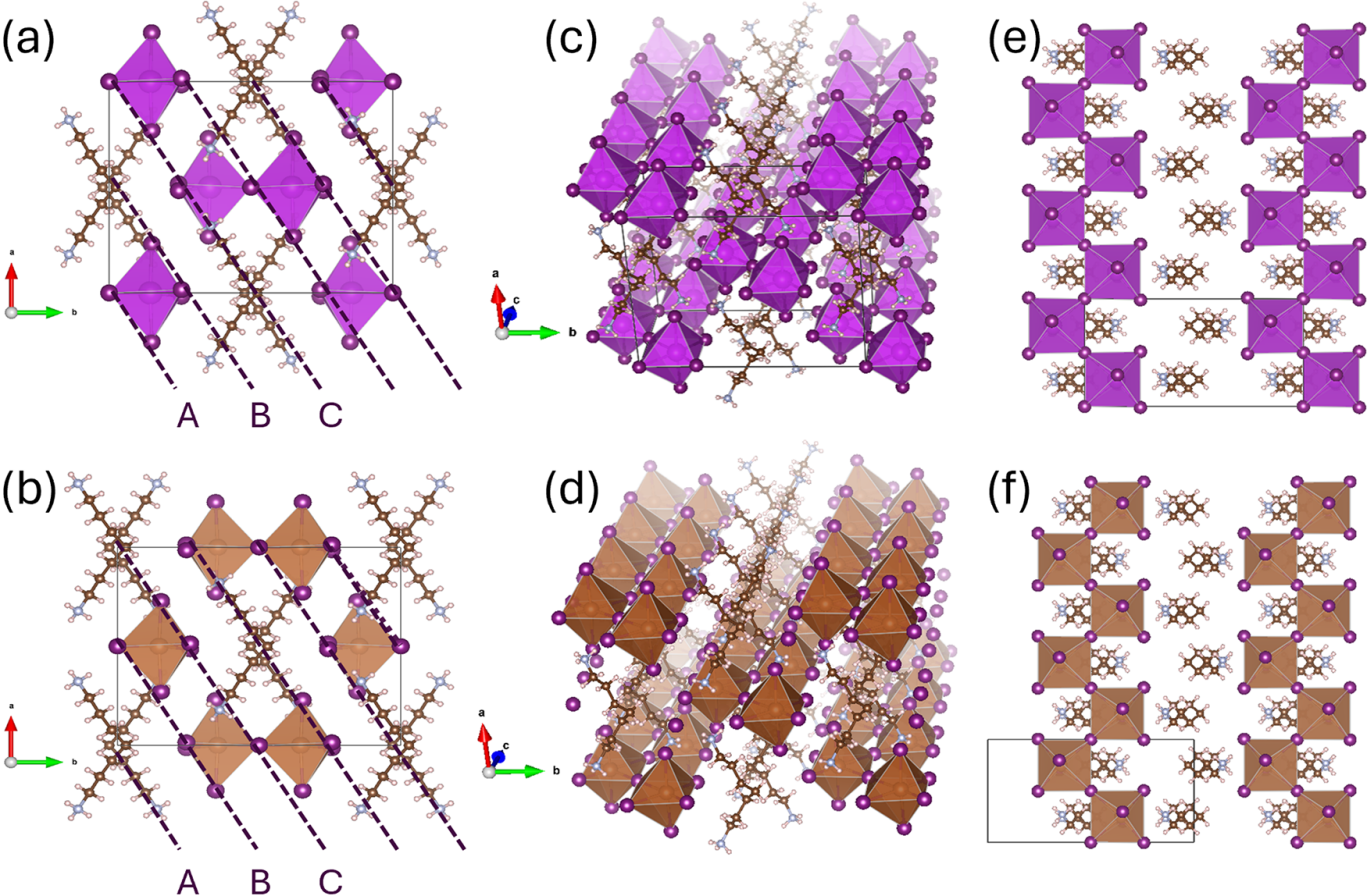
Isostructural crystal structures of (NH_3_(CH_2_)_7_NH_3_)_2_Bi_2_I_10_ (purple octahedra) and (NH_3_(CH_2_)_7_NH_3_)_2_Sb_2_I_10_ (orange octahedra), as determined by single-crystal X-ray diffraction of highly twinned crystals, highlighting key structural features: (a,b) the ideal *ABC* iodide close packing (dashed lines) and the preserved backbone of the (NH_3_(CH_2_)_7_NH_3_)^2+^ cations concerning the [MI_6_]^3–^ octahedra, (c,d) the corner-connected, corrugated 1D chains of [MI_6_]^3–^ octahedra interspersed with (NH_3_(CH_2_)_7_NH_3_)^2+^ cations, and (e,f) a top-down view of the cross-linked 1D chains of [MI_6_]^3–^ octahedra emphasizing the staggering of corrugated 1D chains between octahedral layers.

**Table 1:** (NH_3_(CH_2_)_7_NH_3_)_2_Bi_2_I_10_ and (NH_3_(CH_2_)_7_NH_3_)_2_Sb_2_I_10_ Experimental Crystallographic Parameters from Single Crystal Diffraction

	(NH_3_(CH_2_)_7_NH_3_)_2_Bi_2_I_10_	(NH_3_(CH_2_)_7_NH_3_)_2_Sb_2_I_10_
formula wt (g/mol)	1911.64	1777.06
crystal system	monoclinic	monoclinic
space group	*C*2/*c* (No. 15)	*C*2/*c* (No. 15)
*a* (Å)	13.1697(5)	13.0581(7)
*b* (Å)	17.0322(7)	16.6079(7)
*c* (Å)	8.69334(4)	8.6135(4)
α (deg)	90	90
β (deg)	91.267(2)	92.340(2)
γ (deg)	90	90
ν (Å^3^)	1949.53(14)	1866.43(15)
*Z*	2	2
θ range (deg)	3.026–26.461	2.45–26.42
μ (mm^–1^)	16.930	9.733
temperature (K)	300	100
measured reflns	8818	9782
independent reflns	2019	1930
reflns with *I* > 2σ(I)	1990	1819
*R*_int_		0.017
*R*_1_ (F)	0.0213	0.0199
*wR*_2_	0.0567	0.0407
parameters	71	72
goodness-of-fit on *F* ^2^	1.000	1.069
largest diff. peak and hole (e Å^–3^)	1.45 and −1.36	1.03 and −1.48

The resulting single-crystal structures were employed as starting models for the analysis of the room-temperature PXRD data. Quantitative analyses of the PXRD using the Rietveld method are illustrated in Figure 2. PXRD confirms the high phase purity of the bulk powder and a consistent structural model with that of SCXRD. Air stability was confirmed by no change in the product PXRD pattern after air exposure for upward of 6 months. Because of the needle-like structure, a preferred orientation correction was used for the fit of (NH_3_(CH_2_)_7_NH_3_)_2_Sb_2_I_10_ along the {*h*00} family of planes that cleave the center of the octahedra. The primary change in structure for (NH_3_(CH_2_)_7_NH_3_)_2_Sb_2_I_10_ from 100 K (SCXRD, Table 1) to 298 K (room-temperature PXRD, Table 2) is the increase in lattice parameters and corresponding unit cell volume. The extracted structural parameters from room-temperature PXRD of (NH_3_(CH_2_)_7_NH_3_)_2_Sb_2_I_10_ were used for PDF and anion close packing analysis of the ambient sample.

**Figure 2:**
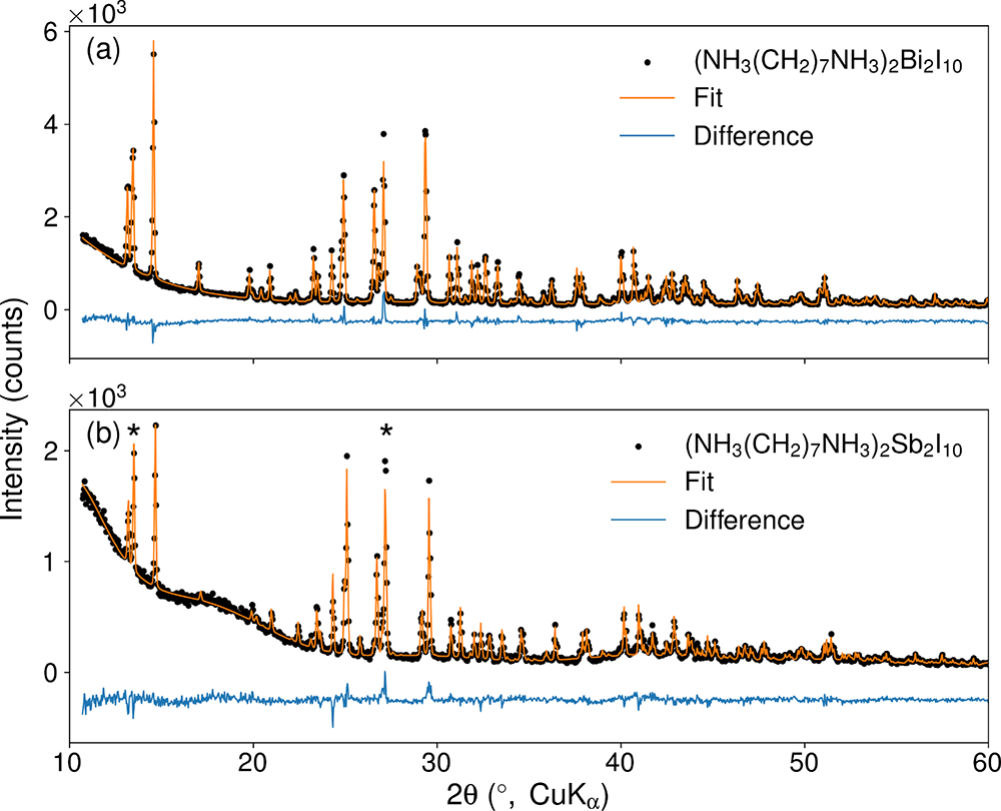
Powder diffraction data collected at room temperature (black circles) alongside the refined structural model obtained through the Rietveld method (orange line) and the corresponding difference curve (blue line, offset below) of (a) (NH_3_(CH_2_)_7_NH_3_)_2_Bi_2_I_10_ and (b) (NH_3_(CH_2_)_7_NH_3_)_2_Sb_2_I_10_. The asterisks (*) in (b) indicate the peaks corresponding to the {*h*00} planes with preferred orientation in (NH_3_(CH_2_)_7_NH_3_)_2_Sb_2_I_10_.

**Table 2:** (NH_3_(CH_2_)_7_NH_3_)_2_Bi_2_I_10_ and (NH_3_(CH_2_)_7_NH_3_)_2_Sb_2_I_10_ Experimental Crystallographic Parameters for Structures Solved from Rietveld Refinement

	(NH_3_(CH_2_)_7_NH_3_)_2_Bi_2_I_10_	(NH_3_(CH_2_)_7_NH_3_)_2_Sb_2_I_10_
formula wt (g/mol)	1955.77	1777.23
temperature (K)	298	298
crystal system	monoclinic	monoclinic
space group	*C*2/*c* (No. 15)	*C*2/*c* (No. 15)
*a* (Å)	13.1784(7)	13.1343(6)
*b* (Å)	17.0290(2)	16.8856(3)
*c* (Å)	8.7045(1)	8.6609(7)
β (deg)	91.3143(1)	91.5783(7)
*V* (Å^3^)	1952.92(2)	1920.11(8)
*Z*	2	2
χ^2^	1.88	1.47
*R*_p_	6.95%	5.52%
*R*_wp_	9.24%	7.76%
preferred orientation	N/A	{*h*00}

From comparison of the anion substructures, (NH_3_(CH_2_)_7_NH_3_)_2_Bi_2_I_10_ exhibits a more ideally close-packed anion substructure. Here, an ideal iodine close packed structure was generated using a *Fm*3̅*m* unit cell with lattice parameters adjusted such that the distance between close packed layers matched the average bond lengths of the close-packed iodine atoms in (NH_3_(CH_2_)_7_NH_3_)_2_Bi_2_I_10_ and (NH_3_(CH_2_)_7_NH_3_)_2_Sb_2_I_10_ as 4.388(5) Å and 4.340(7) Å respectively; this was then transformed to the same setting as the titled compounds. These overlapped structures are illustrated in Figure 3. Here, the Bi-derived structure has consistently lower differences in iodine positions and lattice deformation strain, reported as *d*_max_ = 0.6229 Å, *d*_avg_ = 0.3398 Å and *s* = 0.0221, compared to that of Sb­(III) with *d*_max_ = 0.6984 Å, *d*_avg_ = 0.3643 Å and *s* = 0.0238 (following the definitions of COMPSTRU^57^). A previously reported structure with the same organic cation, (NH_3_(CH_2_)_7_NH_3_)_2_Sn_3_I_10_,^44^ exhibits a 3D architecture, where the atomic radius of Sn­(II) (1.0 Å) may promote ideal packing of the inorganic framework and accommodation of the organic cation without reducing dimensionality, where Bi­(III) (1.03 Å) is too large and Sb­(III) (0.76 Å) is too small, though Bi­(III) is closer to this ideal size.^26^

**Figure 3:**
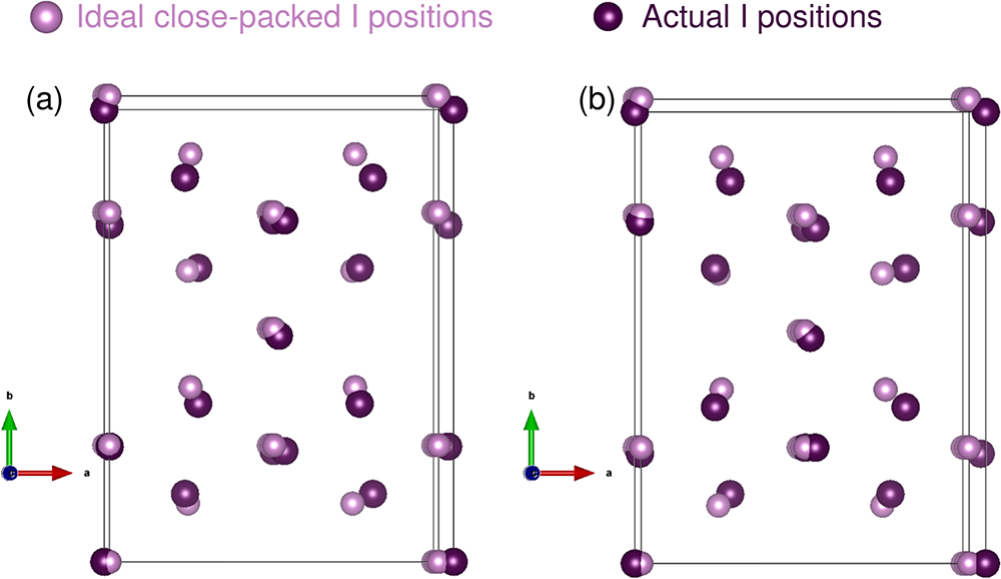
Unit cells of (a) (NH_3_(CH_2_)_7_NH_3_)_2_Bi_2_I_10_ and (b) (NH_3_(CH_2_)_7_NH_3_)_2_Sb_2_I_10_ displaying only iodide anions in experimental crystal structures (dark purple spheres) and in calculated ideal anion close-packed crystal structures (light purple spheres) overlaid to highlight slight differences in iodide close packing.

### Pair Distribution Function Analysis

X-ray total scattering experiments and the subsequent pair distribution function (PDF) analyses of (NH_3_(CH_2_)_7_NH_3_)_2_Bi_2_I_10_ and (NH_3_(CH_2_)_7_NH_3_)_2_Sb_2_I_10_ closely match the crystallographic models. Illustrated in Figure 4, (NH_3_(CH_2_)_7_NH_3_)_2_Bi_2_I_10_ required refinement of a scale factor, lattice parameters (*a*, *b*, *c*, and β), and an *r*-dependent peak narrowing at low *r* to account for correlated atomic motion (δ_2_,^78^ *R*_wp_ = 22%). For (NH_3_(CH_2_)_7_NH_3_)_2_Sb_2_I_10_, isotropic thermal displacement parameters grouped by atom type (*U*(Sb), *U*(I), *U*(organic)) required refinement to obtain an equally good fit (*R*_wp_ improves from 33 to 21%). This may indicate that the Sb­(III) derived structure is locally more flexible compared to that of Bi­(III). For both compounds, no unmodeled peaks or peak shoulders are used in the average, crystallographic structure obtained from SCXRD or PXRD analysis.

**Figure 4:**
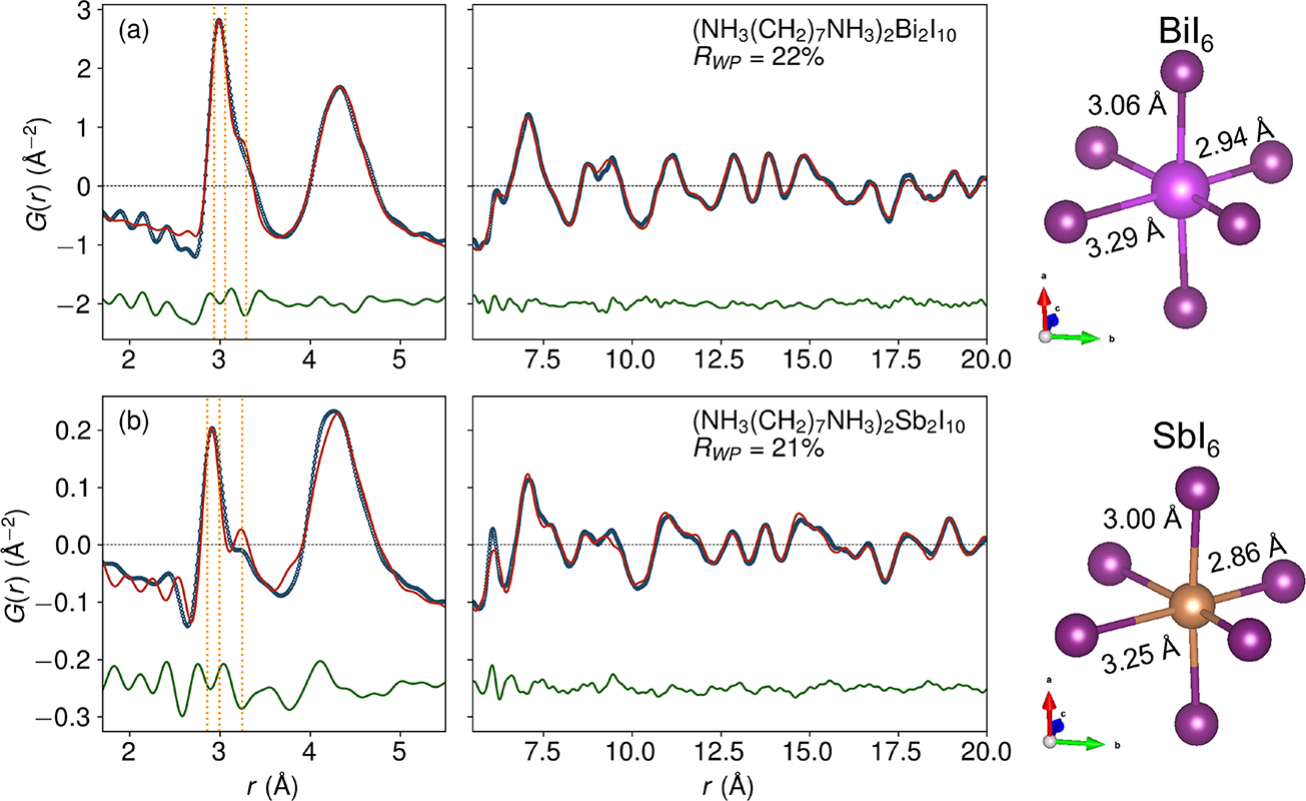
Pair distribution function (PDF) analyses derived from X-ray total scattering data (blue circles) alongside the refined structural model (red line) and the corresponding difference curve (green line, offset) of (a) (NH_3_(CH_2_)_7_NH_3_)_2_Bi_2_I_10_ and (b) (NH_3_(CH_2_)_7_NH_3_)_2_Sb_2_I_10_. The *r*-axis is split to highlight local coordination geometry (1.7 Å≤ *r* ≤ 5.5 Å) and the extended structure (5.5 ≤ *r* ≤ 20 Å). The vertical orange dotted lines are representative of the three average M-I bond lengths in the distorted [MI_6_]^3–^ octahedra (M = Bi^3+^, Sb^3+^), pictured on the right.

The asymmetric peaks centered at ≈3 Å in the M-I bonding region show similar modes of octahedral distortion in [BiI_6_]^3–^ and [SbI_6_]^3–^ and similar long-range disorder. The M­(III) cation is shifted toward the in-plane organic cation, producing a pair of equatorial bonds significantly longer than the axial bond. The broader peak shoulder in (NH_3_(CH_2_)_7_NH_3_)_2_Sb_2_I_10_ at low Å is indicative of a higher magnitude of octahedral distortion, where the equatorial bond lengths are less symmetric relative to the perfect *Fm*3̅*m* perovskite. This increased distortion of the Sb-based octahedron is consistent with increased stereochemical activity of a lone pair moving up the column in the periodic table.^22^ The broader peak shoulder in (NH_3_(CH_2_)_7_NH_3_)_2_Sb_2_I_10_ at high Åmay be indicative of a more long-range distortion of the inorganic framework.

### Optical Properties

Diffuse reflectance spectroscopy was performed for the title compounds and analyzed via the Kubelka–Munk transformation: *k*/*s* = (1 – *R*_*∞*_)^2^/(2*R*_*∞*_), where *k* is the absorption coefficient, *s* is the scattering coefficient, and *R*_*∞*_ represents the measured diffuse reflectance. The pseudoabsorbance is then represented as *k*/*s*, as shown in Figure 5 (at various temperatures). The pseudoabsorbance is normalized to (*k*/*s*)^2^ and (*k*/*s*)^1/2^ to linearize the curves associated with direct and indirect bandgaps respectively, assuming parabolic band shapes, and the tangent line of each curve is extrapolated to determine the bandgap energy.^79^

**Figure 5:**
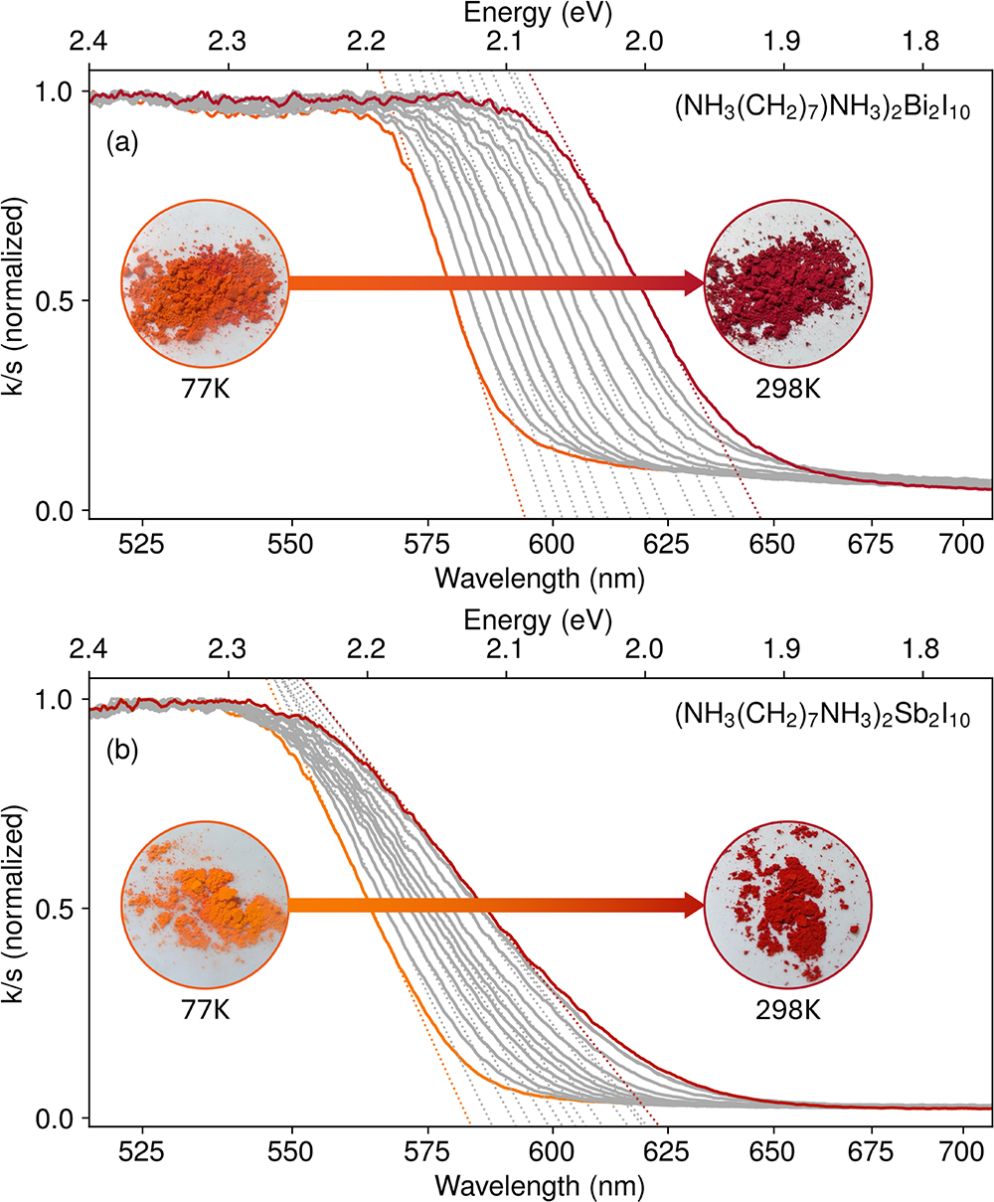
Normalized pseudoabsorbance (*k*/*s*) obtained from diffuse reflectance spectroscopy of (a) (NH_3_(CH_2_)_7_NH_3_)_2_Bi_2_I_10_ and (b) (NH_3_(CH_2_)_7_NH_3_)_2_Sb_2_I_10_ upon warming from 77 to 298 K, where the light orange curves correspond to liquid nitrogen cooled bulk powder, the dark red curves correspond to the room temperature bulk powder, and the gray curves are the pseudoabsorbance upon ambient warming of the sample, taken every second (some curves are excluded for clarity). The onset of visible absorption of energy is emphasized by tangent lines (dotted lines) of each curve.

The estimated optical gap, direct bandgap, and indirect bandgap values for (NH_3_(CH_2_)_7_NH_3_)_2_Bi_2_I_10_, obtained from Tauc analysis, are 1.920(3), 1.972(3), and 1.845(3) eV at room temperature, respectively (Figure 5a, dark red curve). These values are lower than those of (NH_3_(CH_2_)_7_NH_3_)_2_Sb_2_I_10_ at 1.995(3) eV, 2.077(4) eV, and 1.879(7) eV (Figure 5b, dark red curve), contrary to what general design principles might predict.^8,42^ The metal *s*-orbital, a significant contributor to the valence band of these materials, sits at a lower energy in Bi­(III), which should widen the bandgap; however, the observed bandgaps are very close in magnitude, with a slightly lower gap observed with the Bi-based compound. We suggest that the contradiction to this is due to anion close-packing in the inorganic framework. Iodine 5*p* orbitals primarily contribute to the valence band of these materials, and their role may dominate the electronic properties of these materials, which has been previously examined as a function of defect states in other vacancy-ordered perovskites.^43^ The less ideal iodine close packing in (NH_3_(CH_2_)_7_NH_3_)_2_Sb_2_I_10_ would reduce orbital overlap in the electronic structure and widen the band gap, supporting the unexpected increase in bandgaps relative to (NH_3_(CH_2_)_7_NH_3_)_2_Bi_2_I_10_. The decreased dimensionality of these materials is also reflected in bandgap widening relative to the 3D Sn­(III) analog, (NH_3_(CH_2_)_7_NH_3_)_2_Sn_3_I_10_,^44^ with an optical band gap = 1.86(1) eV, due to decreased orbital overlap of the 1D corner-connected chains.

(NH_3_(CH_2_)_7_NH_3_)_2_Bi_2_I_10_ and (NH_3_(CH_2_)_7_NH_3_)_2_Sb_2_I_10_ exhibit a brilliant color change from dark red to bright orange upon cooling with liquid nitrogen, as shown in Figure 5. This color change was initially observed when cooling with liquid nitrogen during SCXRD characterization of (NH_3_(CH_2_)_7_NH_3_)_2_Sb_2_I_10_, as room-temperature SCXRD data acquisition did not elucidate organic atomic positions; this contrasts with (NH_3_(CH_2_)_7_NH_3_)_2_Bi_2_I_10_, in which all atom positions could be elucidated at room temperature. Together, these results suggest more organic motion in (NH_3_(CH_2_)_7_NH_3_)_2_Sb_2_I_10_ at room temperature than that in the Bi analogue. The less ideal iodine packing of (NH_3_(CH_2_)_7_NH_3_)_2_Sb_2_I_10_ and a higher degree of octahedral distortion may allow more flexibility in the organic cation at modest temperatures that indirectly but significantly impact the electronic properties.

To further investigate this, the diffuse reflectance spectroscopy (DRS) of (NH_3_(CH_2_)_7_NH_3_)_2_Bi_2_I_10_ and (NH_3_(CH_2_)_7_NH_3_)_2_Sb_2_I_10_ at liquid nitrogen (77 K) and room temperature (298 K) was examined to further analyze differences in the interaction of the organic cation and inorganic framework, as shown in Figure 5. The difference in pseudoabsorbance (*k/s*) as a function of warming shows a stark difference in color, absorption edge shift, and shape of the onset of visible light absorption between (NH_3_(CH_2_)_7_NH_3_)_2_Bi_2_I_10_ and (NH_3_(CH_2_)_7_NH_3_)_2_Sb_2_I_10_. In (NH_3_(CH_2_)_7_NH_3_)_2_Bi_2_I_10_, there is a smooth redshift in the absorption edge from 2.088 at 77 K to 1.920 eV at 298 K, where a sharp onset of absorption is maintained, evidenced by relatively parallel tangent lines, suggestive of trivial thermal expansion. Contrastingly, in (NH_3_(CH_2_)_7_NH_3_)_2_Sb_2_I_10_, the redshift from 2.128 at 77 K to 1.995 eV at 298 K is much broader, evidenced by a broadening of the onset of absorption and a changing slope of the extrapolated tangent lines. This suggests an increase in the number of allowed transitions from the valence band to the conduction band and a broader range of visible light absorption, as would happen from dynamical exploration of local configurations in (NH_3_(CH_2_)_7_NH_3_)_2_Sb_2_I_10_, as observed in hybrid layered perovskites with more dynamic organic cations.^80^ We attribute these differences in temperature-dependent absorption to the decrease in iodine close packing that may permit higher flexibility of the organic cation in (NH_3_(CH_2_)_7_NH_3_)_2_Sb_2_I_10_ that is emphasized upon an increase in temperature.

### Density Functional Theory Calculations

Density functional theory (DFT) calculations report a direct and indirect bandgap of 2.179 and 2.247 eV for (NH_3_(CH_2_)_7_NH_3_)_2_Bi_2_I_10_ and 2.130 and 2.157 eV for (NH_3_(CH_2_)_7_NH_3_)_2_Sb_2_I_10_, respectively. The discrepancy in these values compared to those of the experimental is likely due to organic cation dynamics that are not fully captured or excitonic effects, as discussed in the Microwave Conductivity section. From the DFT-derived dielectric tensors and effective masses, Fröhlich polaron coupling constants of α = 5.68 and 5.17 were calculated for the Bi and Sb analogues, respectively, indicating strong electron–phonon coupling in both materials. Within Feynman’s variational path-integral framework, this yields Hellwarth polaron mobilities of 2.72 cm^2^ V^–1^ s^–1^ (Bi) and 3.79 cm^2^ V^–1^ s^–1^ (Sb) at 300 K, with corresponding polaron radii of ∼2.14 nm and ∼2.29 nm and polaron mass enhancements of 172% and 147% over the bare band mass. The larger polaron coupling and heavier polaron mass in the Bi analogue are consistent with its larger effective carrier masses (harmonic mean *m*_e_^*^ = 1.23 *m*_e_, *m*_h_^*^ = 5.89 *m*_e_) compared to the Sb analogue (*m*_e_^*^ = 1.05 *m*_e_, *m*_h_^*^ = 1.62 *m*_e_). Within effective mass theory, the Wannier–Mott exciton binding energies are estimated at 908 meV (Bi) and 521 meV (Sb), with correspondingly small exciton Bohr diameters of 0.41 and 0.68 nm, reflecting the large carrier masses and moderate dielectric screening (ε_∞_ ≈ 3.9–4.1) characteristic of these low-dimensional hybrid perovskites. These large binding energies and subnanometre Bohr radii are consistent with the pronounced excitonic features observed experimentally. The electronic structure shows relatively flat dispersion bands due to the decreased dimensionality of the material, shown in Figure 6, compared to that of the 3D (NH_3_(CH_2_)_7_NH_3_)_2_Sn_3_I_10_ with some dispersive bands.^44^ The slight increase in dispersion along the Γ → *A* and *L* → *V* points in the Brillouin zone corresponds to the real-space *c*-vector along the octahedral chains, where there would be the most orbital overlap in the structure. Partial densities of states also reflect that typical of hybrid halide perovskites, with I 5*p* orbitals dominating the frontier valence orbitals and the M­(III) *p*-orbitals (M = Bi^3+^, Sb^3+^) dominating the frontier conduction band orbitals.

**Figure 6:**
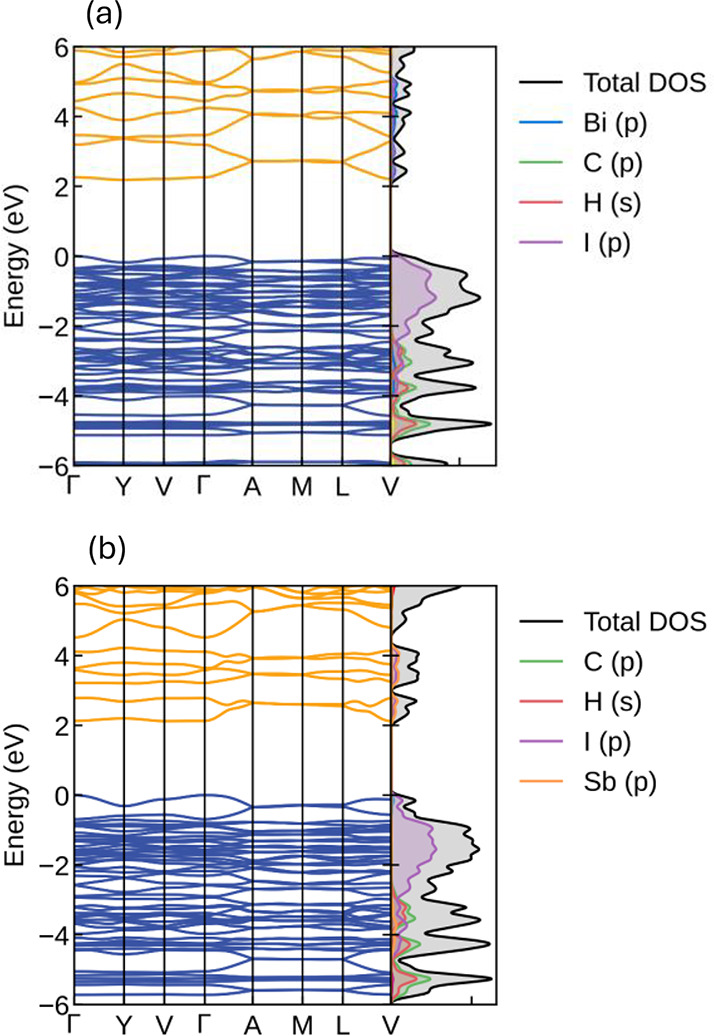
Electronic band structures and full and partial densities of states of (NH_3_(CH_2_)_7_NH_3_)_2_Bi_2_I_10_ (a) and (NH_3_(CH_2_)_7_NH_3_)_2_Sb_2_I_10_ (b). The valence bands and conduction bands are represented by blue and orange lines respectively as a function of critical points in the Brillouin zone and show relatively flat dispersion. The full density of states is represented in gray and is decomposed into orbital projections.

### Microwave Conductivity

Dark and time-resolved microwave conductivity measurements were performed to evaluate the equilibrium and excited-state electronic properties of (NH_3_(CH_2_)_7_NH_3_)_2_Bi_2_I_10_ and (NH_3_(CH_2_)_7_NH_3_)_2_Sb_2_I_10_, though time-resolved measurements were only conducted for the latter. In these AC measurements, conductivity is a complex quantity that can be expressed either as a complex conductivity, a complex permittivity, or some combination of the two, as these are not distinguishable material properties a priori. This equivalence is expressed by the relation σ = ϵ_r_^″^(ωϵ_0_) + *i*ϵ_r_′(ωϵ_0_), and we choose to express the results as a real permittivity and a real conductivity. The real permittivity of (NH_3_(CH_2_)_7_NH_3_)_2_Sb_2_I_10_, 13.85, was higher than that of (NH_3_(CH_2_)_7_NH_3_)_2_Bi_2_I_10_, 11.27, and indicates relatively high polarizability in both materials. Both compounds have similar equilibrium real conductivities 1.53(20) × 10^–3^ S/cm for Bi vs 2.89(18) × 10^–3^ S/cm for Sb, compiled in Table 3.

**Table 3:** Electronic Properties of (NH_3_(CH_2_)_7_NH_3_)_2_Bi_2_I_10_ as Determined by Time-Resolved Microwave Conductivity (TRMC)

	(NH_3_(CH_2_)_7_NH_3_)_2_Bi_2_I_10_
K-factor	–4741.39
real dielectric constant	11.27
frequency (GHz)	9.6
conductivity (S/cm)	1.53(20) × 10^–3^
mobility (cm^2^/V/s)	2.5 × 10^–4^
doping density (cm^–3^)	3.82 × 10^19^
density of the powder (*a*) [g/cm^–3^]	1.12
unit cell density (*b*) [g/cm^–3^]	3.326
filling fraction (ff = *a*/*b*)	0.337(43)

Time-resolved conductivity (TRMC) measurements were performed on (NH_3_(CH_2_)_7_NH_3_)_2_Bi_2_I_10_ at two excitation wavelengths, 355 and 600 nm, and a range of excitation fluences, 9 × 10^13^ to 6 × 10^15^; Figure 7. These measurements provide nanosecond-resolved real photoconductivity, which we express as the product of carrier yield (ϕ) and the sum of electron and hole mobilities (Σμ).

**Figure 7:**
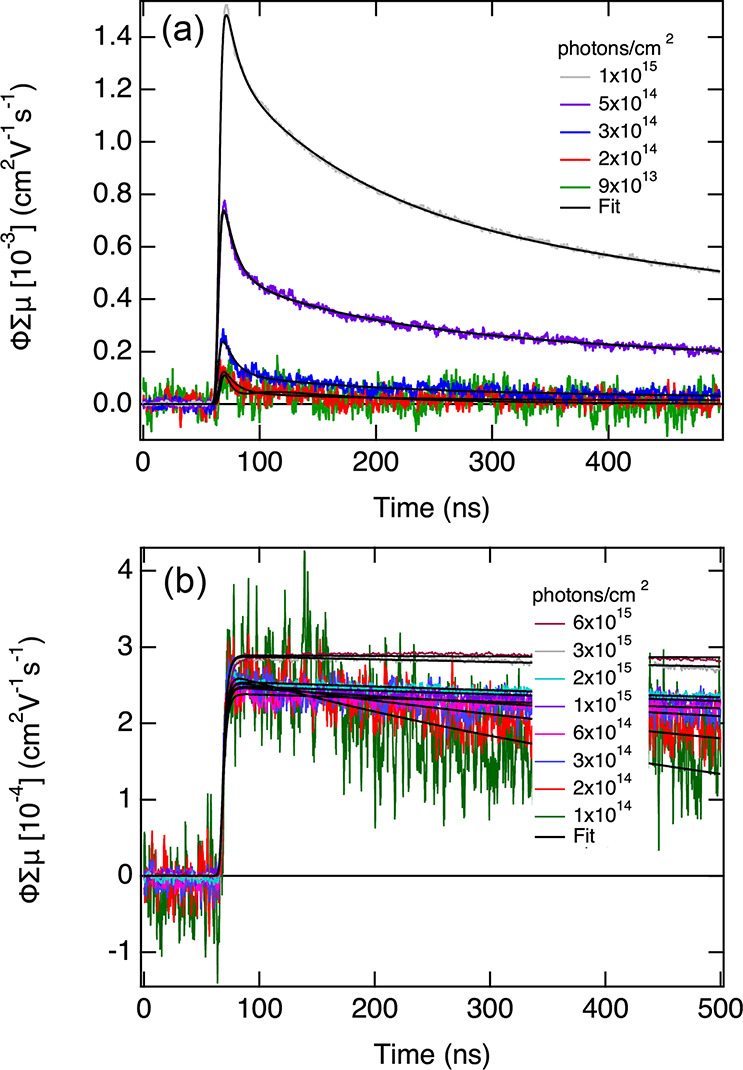
Time-dependent yield-mobility product (ΦΣμ) at a frequency of 9.60 GHz for two excitation wavelengths: (a) 355 nm and (b) 600 nm. Different color traces represent varying light fluences, ranging from 10^13^ to 10^15^ photons/cm^2^. The black lines indicate the fitted curves corresponding to the measured transients of (NH_3_(CH_2_)_7_NH_3_)_2_Bi_2_I_10_.

The carrier dynamics of (NH_3_(CH_2_)_7_NH_3_)_2_Bi_2_I_10_ show an unusual dependence of lifetime and yield-mobility on excitation fluence and wavelength; Figure 8. The increase in carrier mobility with fluence suggests that it is not limited by carrier–carrier scattering. The fluence dependence of carrier lifetime suggests that nonradiative recombination, primarily driven by defect states and interactions with dark carriers, dominates the relaxation dynamics in (NH_3_(CH_2_)_7_NH_3_)_2_Bi_2_I_10_. Upon excitation, defect states close to band edges are filled and extend the lifetimes of the remaining free carriers as a function of increasing fluence. The wavelength-dependent behavior is unexpected, as photoconductivity and emission are typically independent of excitation energy due to ultrafast relaxation to the band edge. The higher energy 355 nm excitation may promote saturation of filled trap states, where mobility is increased for some duration until relaxing to the band edge and exhibiting behavior similar to that at 600 nm. This is seen in Figure 7, where at 355 nm excitation with fluences below 5 × 10^14^, the mobility approaches that of 600 nm excitation, ≈2 × 10^–4^ around 150 ns. The higher absorption cross-section at 355 nm may also produce a higher concentration of carriers at the surface of the material, promoting carrier recombination and lowering lifetimes. Higher penetration into the bulk material at 600 nm, with a lower absorption cross section, reduces carrier concentration and extends carrier lifetimes. The deviation from expected behavior underscores the complex interaction of carrier generation, relaxation, and recombination in (NH_3_(CH_2_)_7_NH_3_)_2_Bi_2_I_10_, suggesting the presence of shallow traps that strongly influence the photophysical properties of the material.

**Figure 8:**
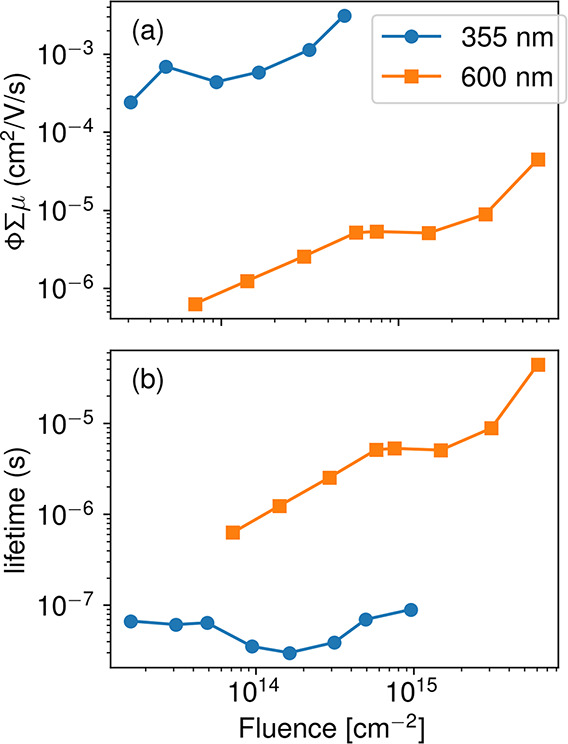
(a) Yield–mobility product (cm^2^ V^–1^ s^–1^) as a function of fluence (cm^–2^) under 355 and 600 nm excitation and (b) carrier lifetimes (s) as a function of fluence (cm^–2^) for the same materials under 355 and 600 nm excitation, illustrating the photoconductivity-excitation wavelength dependence for (NH_3_(CH_2_)_7_NH_3_)_2_Bi_2_I_10_.

The equilibrium carrier concentration of (NH_3_(CH_2_)_7_NH_3_)_2_Bi_2_I_10_ can be calculated using the mobility and intrinsic conductivity values (see Table 3) obtained from dark and time-resolved microwave conductivity measurements using the relation σ = *en*μ, assuming a photogenerated carrier yield of unity (Φ = 1). However, we illustrate below that this is a poor assumption in these materials, which exhibit confined electronic structures. Assuming ϕ = 1 leads to an unusually high calculated carrier density of 3.82 × 10^19^ cm^–3^ in (NH_3_(CH_2_)_7_NH_3_)_2_Bi_2_I_10_, on the order of self-p-type doping in Sn­(II)-based perovskites that promote metallic behavior.^81,82^

It is clear that assuming a perfect photon-to-carrier yield (Φ = 1) artificially reduces the interpreted carrier mobility and inflates the carrier concentration. A deeper consideration of the probable carrier yield is thus called for. Using the Saha equation,

$$\frac{\alpha^{2}}{1-\alpha }=\frac{1}{N_{\mathrm{tot}}}{\left(\frac{2\pi k_{B}T\mu }{h^{2}}\right)}^{3/2}\exp\left(\frac{-E_{b}}{k_{B}T}\right)$$ 3

One can calculate the ratio of free carriers to the photoexcitation density, α, as a function of exciton binding energy (where μ and *E*_b_ are the exciton reduced mass and binding energy; *T* is the absolute temperature).^83^ Using the DFT calculated electron and hole effective masses to estimate the exciton reduced mass and exciton binding energy (*E*_b_) of 908 meV, α is calculated as 2.68 × 10^–8^, much smaller than the assumed Φ = 1. The yield mobility product can then be corrected by dividing the measured ΦΣμ by α, resulting in an average corrected yield mobility product 3.43 × 10^2^ cm^2^ V^–1^ s^–1^ that is taken to calculate a carrier concentration of 2.79 × 10^13^ cm^–3^, a much more reasonable value for this electronically confined structure. However, *E*_b_ = 908 meV produces a higher mobility than the Hellwarth polaron mobility estimated from DFT parameters. A structurally analogous pseudo 1D corner-connected perovskite structure^84^ that exhibits a large discrepancy in DFT and experimental *E*_b_ values may explain this behavior. If instead, a reasonable estimate of exciton binding energy (500 meV) is used, we calculate a corrected yield mobility product of 1.28 × 10^–1^ cm^2^ V^–1^ s^–1^ and a carrier concentration of 7.45 × 10^16^ cm^–3^ at 600 nm. These calculations point to a plausible range of values for the mobility and carrier density, but their sensitivity toward the unknown exciton binding energy prohibits greater precision. No strong photoluminescence suggestive of exciton formation was observed at 300 K, making it extra challenging to provide experimental estimates of the exciton binding energy.

Based on structural and optoelectronic characterization of these materials, we posit that correlating the size of the organic diammonium cation to the inorganic framework, particularly the anion substructure, provides insight into guided crystal chemical approaches to target specific chemical structures and their corresponding optoelectronic properties, as presented in Figure 9. The distribution of the N–N distances for (NH_3_(CH_2_)_n_NH_3_)^2+^ molecules in experimental crystal structures queried from the Cambridge Structural Database, shown as a function of *n*, the number of carbons in the diammonium molecules, reveals a systematic and conserved trend. Taken together with specific data points related to gaps in the inorganic frameworks, one can predict which diammonium organic cation choice is a “best fit” to promote specific structural archetypes. Examination of the N–N size distributions provides insight into the different conformations the molecule may take in previously synthesized materials, ranging from a linear to a more bent shape as a function of carbon length (as in (NH_3_(CH_2_)_7_NH_3_)­Bi_2_I_10_).^85^ An organic cation similar in size to a given [BX_6_] octahedra can be used to predict how it may incorporate into the structure, where a size “match” may promote higher electronic dimensionality based on retained anion close packing, but one too small or too large may reduce structural dimensionality. This brings an analogous approach to the ellipsoidal analysis of coordination polyhedra for inorganic polyhedra.^86^ Together, these provide a framework for identifying features needed for accelerating the prediction and discovery of hybrid materials.

**Figure 9:**
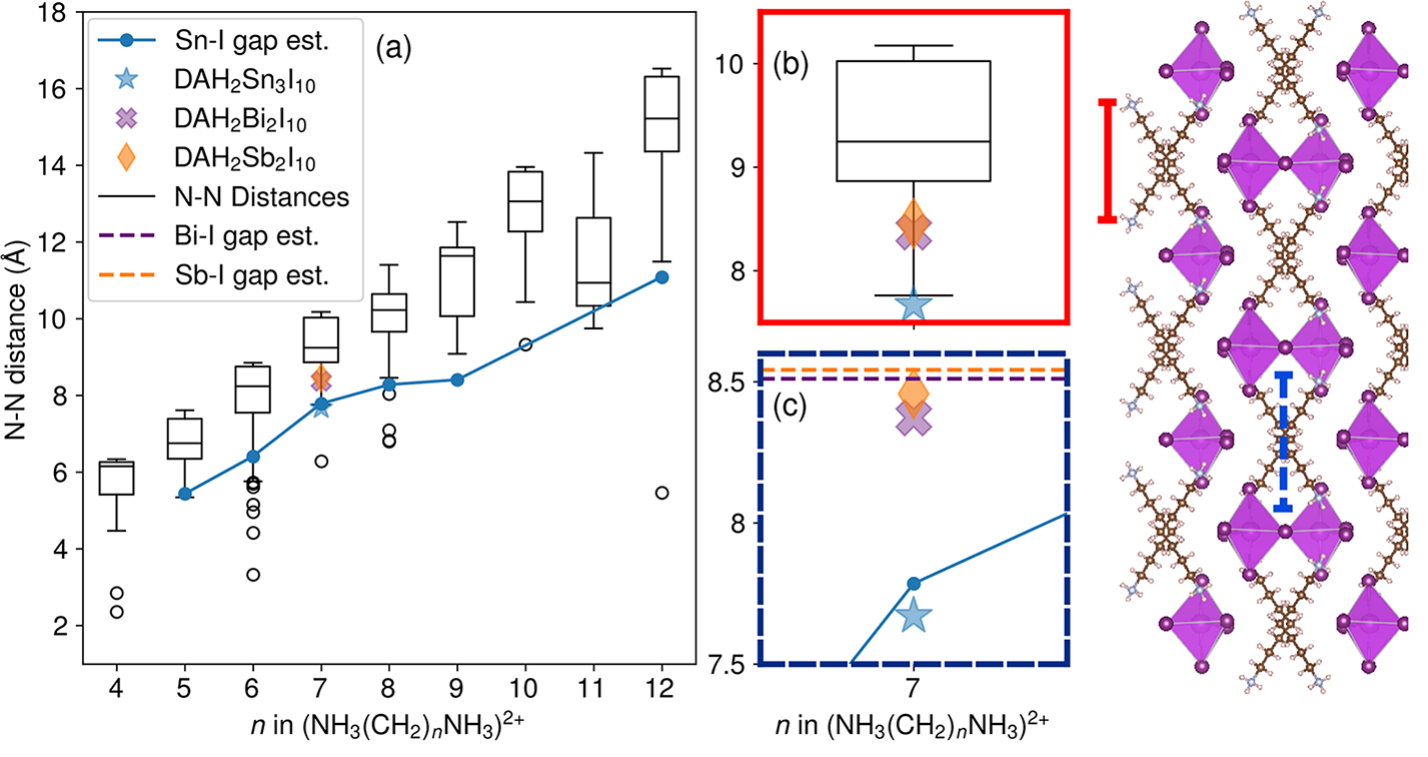
(a) Box plot of the N–N distance distributions for (NH_3_(CH_2_)_*n*_NH_3_)^2+^ molecules in experimental crystal structures in the Cambridge Structural Database, with emphasis of the experimental N–N distances in (NH_3_(CH_2_)_7_NH_3_)^2+^-M-I compounds (M = Sb^3+^ (orange diamond), Bi^3+^ (purple cross), and Sn^2+^ (blue star)). The outline of each subplot corresponds to measurements represented by the arrows overlaid on the experimental (NH_3_(CH_2_)_7_NH_3_)­Bi_2_I_10_ crystal structure. The inorganic layer gap spacing in diammonium-based *A*SnI_4_ compounds are shown as blue circles.^44^ (b) Subset of the N–N distribution for (NH_3_(CH_2_)_7_NH_3_)^2+^, emphasizing the size of the organic cation and its conformation incorporated into experimental structures. (c) Subset of the N–N distribution for (NH_3_(CH_2_)_7_NH_3_)^2+^ in the structures reported herein, with the estimated interlayer octahedral voids (dotted lines) an organic cation would occupy, emphasizing the close relationship between the anionic framework and incorporated size of a given organic cation.

## Conclusion

This contribution reports the synthesis and structural characterization of vacancy-ordered, pseudo-1-dimensional semiconducting materials (NH_3_(CH_2_)_7_NH_3_)_2_Bi_2_I_10_ and (NH_3_(CH_2_)_7_NH_3_)_2_Sb_2_I_10_. Analysis of the *C*2/*c* crystal structures, characterized by corner-connected octahedral chains interspersed with (NH_3_(CH_2_)_7_NH_3_)^2+^ cations, reveals a nearly close-packed iodide substructure of (NH_3_(CH_2_)_7_NH_3_)_2_Bi_2_I_10_ but imperfect packing in (NH_3_(CH_2_)_7_NH_3_)_2_Sb_2_I_10_. This is supported by larger I–I bond differences of the [BX_6_]^2–^ octahedra via PDF and analysis of the experimental iodine close packing in comparison to a calculated ideal close-packed structure in the same space group. Analysis of liquid nitrogen-cooled and room temperature samples indicates the organic cation in (NH_3_(CH_2_)_7_NH_3_)_2_Sb_2_I_10_ has a stronger influence on structural dynamics, exhibited as a bandgap broadening, decreased structural resolution, and a dramatic color change upon sample warming. From a combination of DFT-based estimates for exciton binding energies and microwave conductivity measurements, (NH_3_(CH_2_)_7_NH_3_)_2_Bi_2_I_10_ exhibits reasonable mobile carriers with long carrier lifetimes, after correcting for such excitonic effects. Data mining of diammonium-containing crystal structures compared to those derived from layered perovskite halides reveals that diammoniumheptane is ideally sized to displace an inorganic octahedron, thus simplifying an approach for accelerating the prediction and discovery of hybrid materials with anion close packing.

## Supplementary Material



## Notes

Dark microwave conductivty resonance curves for (NH_3_(CH_2_)_7_NH_3_)_2_Bi_2_I_10_ and (NH_3_(CH_2_)_7_NH_3_)_2_Sb_2_I_10_; alternative PDF modeling of (NH_3_(CH_2_)_7_NH_3_)_2_Sb_2_I_10_; diffuse reflectance spectroscopy analysis for (NH_3_(CH_2_)_7_NH_3_)_2_Bi_2_I_10_ and (NH_3_(CH_2_)_7_NH_3_)_2_Sb_2_I_10_; and analysis of diammonium containing crystal structures; an interactive notebook (structure_analysis_forPub.ipynb, IPYNB) and compiled data (NNdistances.csv, CSV) provided as Supporting Information at https://github.com/jrneilson/DiammoniumDistances ; crystal structures obtained from DFT-based relaxation (PDF)
