# Supplementary material for: Iodine Close Packing in Hybrid Halide Bismuth(III) and Antimony(III) Semiconductors: (NH3(CH2)7NH3)2Bi2I10 and (NH3(CH2)7NH3)2Sb2I10
Source: Inorg Chem. 2026 Jun 6;65(24):13636–48. doi: 10.1021/acs.inorgchem.6c01909 (PMC13292219; doi:10.1021/acs.inorgchem.6c01909)
Supplement: Supplementary file 1 [file ic6c01909_si_001.pdf]

Supplemental Information: Iodine Close Packing in Hybrid Halide Bismuth(III) and Antimony(III) Semiconductors: $(NH_3(CH_2)_7NH_3)_2Bi_2I_{10}$ and $(NH_3(CH_2)_7NH_3)_2Sb_2I_{10}$

Shelby R. Lane,[†] Dominic Cudjoe Asebiah,[‡] Alexander G. Squires,[¶] Autumn N. Peters,[†] Luke T. MacHale,[†] Lauren Borgia,[†] Mirella K. Villani,[†] David O. Scanlon,[¶] Obadiah G. Reid,[*,§,‖] and James R. Neilson[*,†,‡]

†*Department of Chemistry, Colorado State University, Fort Collins, CO 80523, United States*

‡*School of Materials Science & Engineering, Colorado State University, Fort Collins, CO 80523, United States*

¶*School of Chemistry, University of Birmingham, Edgbaston, Birmingham B15 2TT, U.K.*

§*Renewable and Sustainable Energy Institute, University of Colorado Boulder, Boulder, CO 80303, United States*

‖*Chemistry & Nanoscience Center, National Laboratory of the Rockies, Golden, CO 80401, United States*

E-mail: obadiah.reid@NLR.gov; james.neilson@colostate.edu

Dark Microwave Conductivity (DMC)

Dark conductivity measurements were analyzed using resonance curves (microwave power reflectance vs. frequency) obtained from a brass terminating plate at the position of the cavity iris, an empty capillary sample tube in the cavity, and sample packed into the capillary tube, using modified Teflon ferrules to center the tubes in the cavity in each case.

The resonance curves were fit with a finite-element electromagnetic model[1–4] to extract equilibrium complex dielectric constant, which can also be expressed as a conductivity using the relationship: $\sigma' = \varepsilon_r''(\omega\varepsilon_0)$, $\sigma'' = \varepsilon_r'(\omega\varepsilon_0)$, where ($'$ indicates the real part, and $''$ the imaginary part.). The measured values were corrected to account for sample filling fraction, f, of the tubes using the simplest possible effective medium approximation: that the measured properties of the powder arise from a volume weighted average of air ($\epsilon_r = 1 - 0i$) and the semiconductor. For each sample 4 packing fraction capillaries were packed, and an average filling fraction for each sample, f, was determined. This value was calculated using the measured powder density (ρ_{powder}) and the theoretical crystal density ($\rho_{crystal}$): $f = \rho_{powder}/\rho_{crystal}$. The real part of the sample permittivity is then calculated as: $\varepsilon_r' = \frac{\varepsilon_{powder}'(1-f)}{f}$ and the imaginary part as: $\varepsilon_r'' = \frac{\varepsilon_{powder}''}{f}$.

Packing Fraction Determination

The packing fraction, f, accounts for incomplete filling of the capillary volume by the powder sample. For each sample, four independently packed capillaries, $i = 1, 2, 3, 4$, were prepared. The packing density of each capillary was calculated as,

$$d_{\text{packing},i} = \frac{m_i}{V_i}, \tag{S1}$$

where m_i is the sample mass obtained from the difference between filled and empty capillary weights, and V_i is the sample volume calculated assuming a cylindrical geometry using the measured sample height and the capillary inner diameter. The packing fraction for each capillary was then determined as

$$f_i = \frac{d_{\text{packing},i}}{d_{\text{exp}}}, \tag{S2}$$

where d_{exp} is the theoretically calculated bulk density of the material from Topas.

For each sample, the mean packing fraction was calculated as

$$\bar{f} = \frac{1}{4}\sum_{i=1}^{4} f_i, \tag{S3}$$

and the associated uncertainty was taken as the sample standard deviation of the four capillaries:

$$u_f = \sqrt{\frac{1}{N-1}\sum_{i=1}^{4}\left(f_i - \bar{f}\right)^2}, \tag{S4}$$

where $N = 4$. Because $N - 1 = 3$, this reduces to

$$u_f = \sqrt{\frac{1}{3}\left[(f_1 - \bar{f})^2 + (f_2 - \bar{f})^2 + (f_3 - \bar{f})^2 + (f_4 - \bar{f})^2\right]}. \tag{S5}$$

These sample-specific packing fractions and uncertainties were used in all subsequent corrections of permittivity and conductivity.

Propagation of Uncertainty for Packing Fraction–Corrected Conductivity

The imaginary permittivity and real conductivity were corrected for using the average packing fraction, f, according to,

$$X_{\mathrm{corr}} = \frac{X_{\mathrm{meas}}}{f}, \tag{S6}$$

where X represents either ε'' or σ. The real permittivity was corrected for incomplete packing according to,

$$\varepsilon'_{corr} = \frac{\varepsilon'_m - (1 - f)}{f}, \tag{S7}$$

where ε'_m is the measured real permittivity, f is the mean packing fraction, and ε'_{corr} is the corrected real permittivity.

The source of uncertainty noted for the unscaled (not corrected) permittivities (real $u_{\varepsilon'_\mathrm{m}}$ and imaginary $u_{\varepsilon''_\mathrm{m}}$) is the uncertainty from fitting the resonance curves from an interpolated lookup table from electromagnetic simulations. Packing fraction corrections are further applied later as the largest overall source of uncertainty in these measurements.

The uncertainty in the corrected real permittivity, $u_{\varepsilon'_\mathrm{corr}}$, was calculated using standard propagation of uncertainty for uncorrelated variables and can be written in the standard form,

$$u_{\varepsilon'_\mathrm{corr}} = \frac{1}{|f|}\sqrt{u^2_{\varepsilon'_m} + \left(\frac{\varepsilon'_m - 1}{f}\right)^2 u_f^2}. \quad \text{(S8)}$$

The imaginary permittivity was corrected for incomplete packing according to,

$$\varepsilon''_{corr} = \frac{\varepsilon''_m}{f}, \quad \text{(S9)}$$

where ε''_m is the measured imaginary permittivity, f is the mean packing fraction, and ε''_{corr} is the corrected imaginary permittivity.

The uncertainty in the corrected imaginary permittivity, $u_{\varepsilon''_\mathrm{corr}}$, was calculated using the standard propagation of uncertainty for a quotient of uncorrelated variables, expressed in relative form,

$$u_{\varepsilon''_\mathrm{corr}} = |\varepsilon''_\mathrm{corr}|\sqrt{\left(\frac{u_{\varepsilon''_m}}{\varepsilon''_m}\right)^2 + \left(\frac{u_f}{f}\right)^2}. \quad \text{(S10)}$$

The real component of the raw dark microwave conductivity, σ_m, was calculated from the measured imaginary permittivity according to,

$$\sigma_m = \varepsilon_0 \omega \varepsilon''_m, \quad \text{(S11)}$$

where ε_0 is the vacuum permittivity and ω is the angular frequency determined by

the instrument parameters. Both ε_0 and ω were treated as exact constants. Under this assumption, the uncertainty in the uncorrected real conductivity, u_{σ_m}, propagates directly from the uncertainty in the measured imaginary permittivity,

$$\frac{u_{\sigma_m}}{\sigma_m} = \frac{u_{\varepsilon''_m}}{\varepsilon''_m}. \tag{S12}$$

The uncertainty in the corrected real conductivity, $u_{\sigma_{\text{corr}}}$, was calculated using the standard propagation of uncertainty for a quotient of uncorrelated variables, written in relative form,

$$u_{\sigma_{\text{corr}}} = |\sigma_{\text{corr}}| \sqrt{\left(\frac{u_{\sigma_m}}{\sigma_m}\right)^2 + \left(\frac{u_f}{f}\right)^2}. \tag{S13}$$

These expressions follow directly from the general law of propagation of uncertainty and accounts for contributions from both the measured permittivity and the uncertainty in the packing fraction. All quantities were assumed to be uncorrelated.

Table S1: Raw dielectric properties and measured conductivity prior to f-correction, with uncertainties.

$(NH_3(CH_2)_7NH_3)_2M_2I_{10}$	**Raw ε''_m ± unc.**	**Raw ε'_m ± unc.**	**Raw σ_m (S cm^{-1}) ± unc.**
M = Sb	0.1995 ± 0.00004	5.991 ± 0.0007	0.1122 ± 0.00002
M = Bi	0.0918 ± 0.00003	4.459 ± 0.0006	0.0515 ± 0.00002
**Packing Fractions**			
M = Sb, Averaged packing fraction = 0.3883 ± 0.024			
M = Bi, Averaged packing fraction = 0.3368 ± 0.043			

Table S2: f-corrected dielectric properties and conductivity.

$(NH_3(CH_2)_7NH_3)_2M_2I_{10}$	ε''_{corr} **(imag.)** $\pm$ **unc.**	ε'_{corr} **(real)** $\pm$ **unc.**	σ_{corr} **(S cm^{-1})** $\pm$ **unc.**
M = Sb	0.5139 $\pm$ 0.032	13.85 $\pm$ 0.808	0.0029 $\pm$ 0.00018
M = Bi	0.2726 $\pm$ 0.035	11.27 $\pm$ 1.32	0.0015 $\pm$ 0.00020

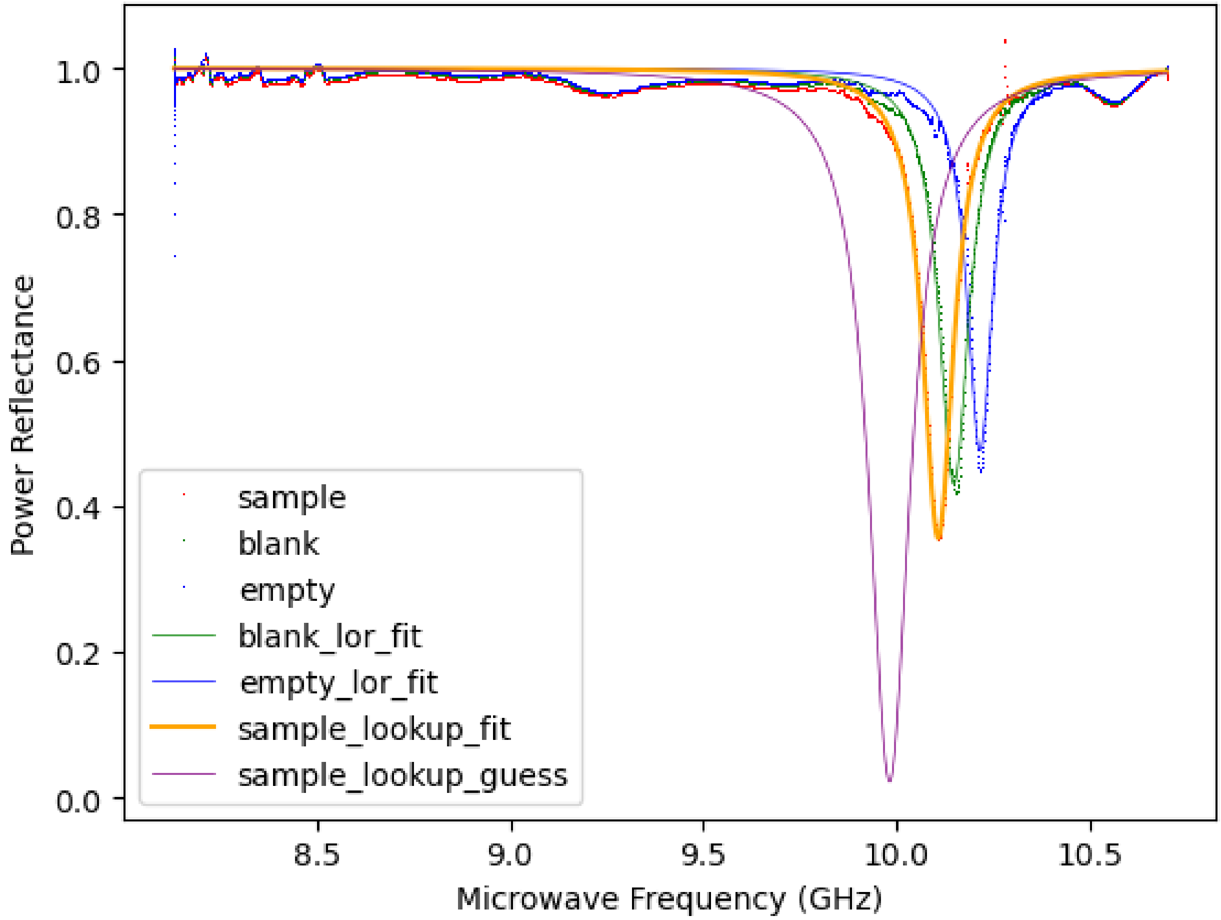

Figure S1: Resonance curves for $(NH_3(CH_2)_7NH_3)_2Bi_2I_{10}$ (red circles) with the x-axis representing frequency (GHz) and the y-axis corresponding to the power reflection coefficient. The orange line indicates Lorentzian fit constrained by electromagnetic simulations of cavity behavior. These simulations were utilized to determine the equilibrium conductivities and relative dielectric constants of $(NH_3(CH_2)_7NH_3)_2Bi_2I_{10}$.

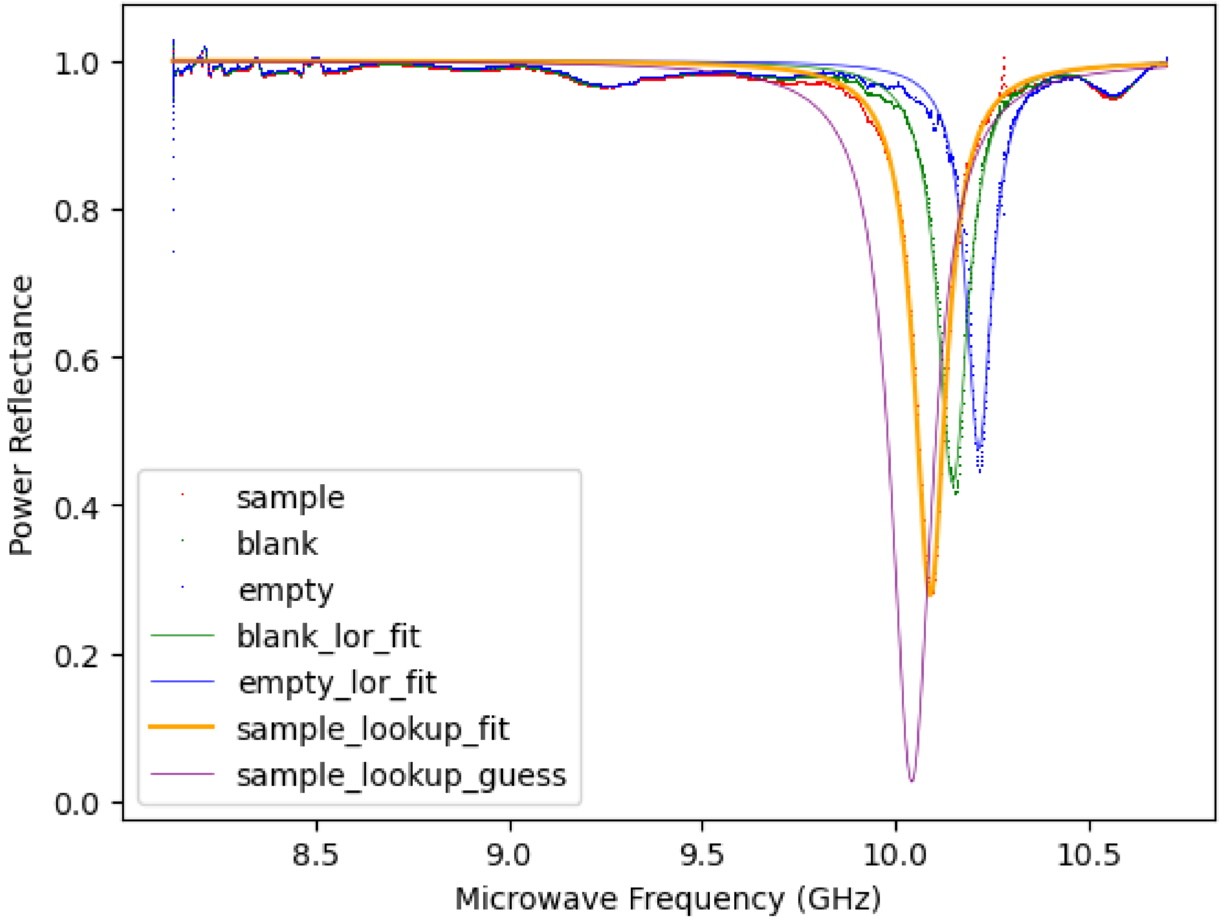

Figure S2: Resonance curves for $(NH_3(CH_2)_7NH_3)_2Sb_2I_{10}$ (red circles) with the x-axis representing frequency (GHz) and the y-axis corresponding to the power reflection coefficient. The orange line indicates Lorentzian fit constrained by electromagnetic simulations of cavity behavior. These simulations were utilized to determine the equilibrium conductivities and relative dielectric constants of $(NH_3(CH_2)_7NH_3)_2Sb_2I_{10}$.

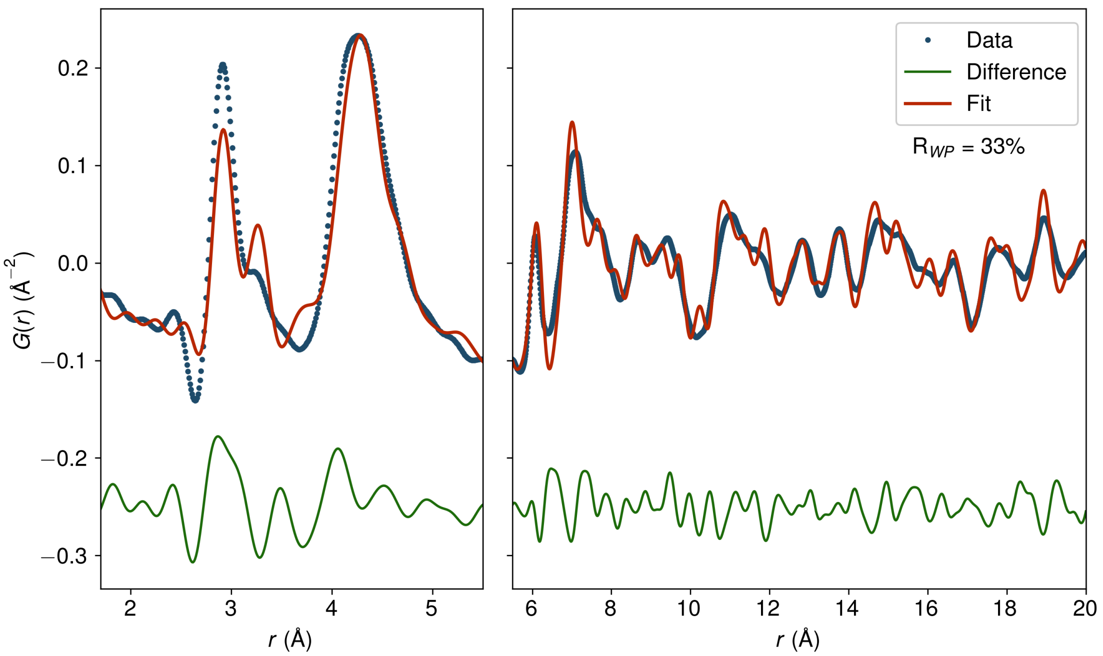

Figure S3: Pair distribution function (PDF) derived from X-ray total scattering data (blue circles) alongside the refined structural model (red line) and the corresponding difference curve (green line, offset) of $(NH_3(CH_2)_7NH_3)_2Sb_2I_{10}$. The x-axis is split to highlight local coordination geometry ($1.7 \leq r \leq 5.5$ Å) and the extended structure ($5.5 \leq r \leq 20$ Å). The vertical orange dotted lines are representative of the three average M-I bond lengths in the distorted $[MI_6]^{3-}$ octahedra (M = Bi^{3+}, Sb^{3+}), pictured on the right.

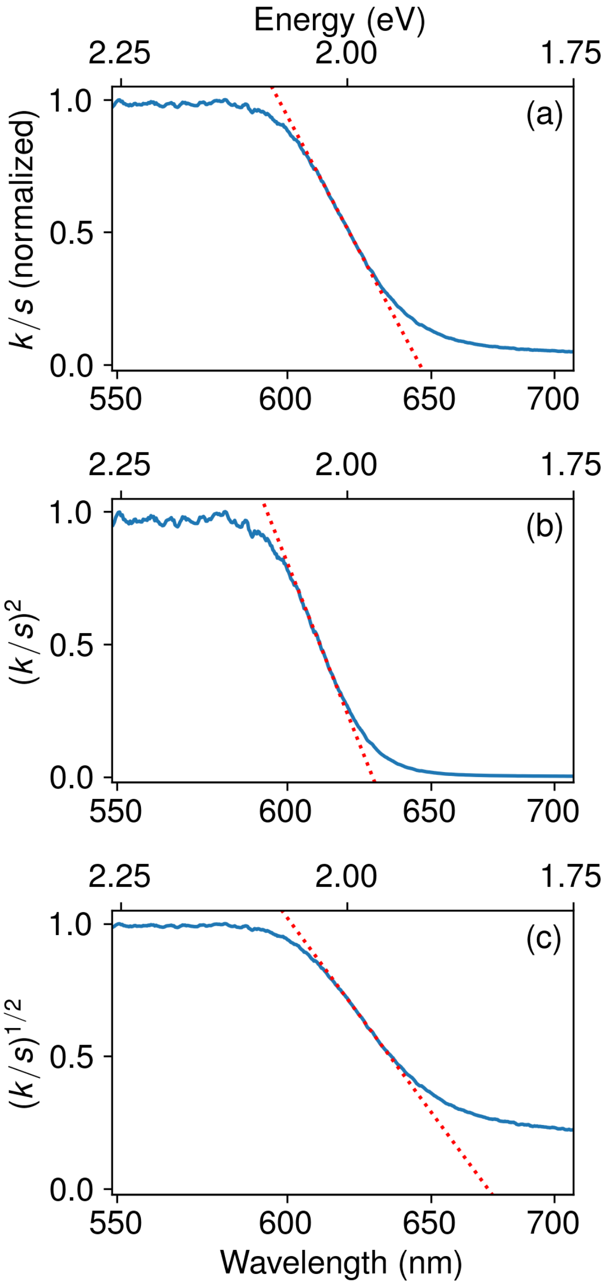

Figure S4: Diffuse reflectance spectra in the UV-visible range analyzed using different methods: (a) applying the Kubelka-Munk transformation to obtain pseudo-absorbance ($F(R) = k/s$), (b) squaring $F(R)$ to linearize direct band gaps associated with bands exhibiting parabolic dispersion, and (c) taking the square root of $F(R)$ to linearize indirect band gaps with parabolic dispersion for $(NH_3(CH_2)_7NH_3)_2Bi_2I_{10}$

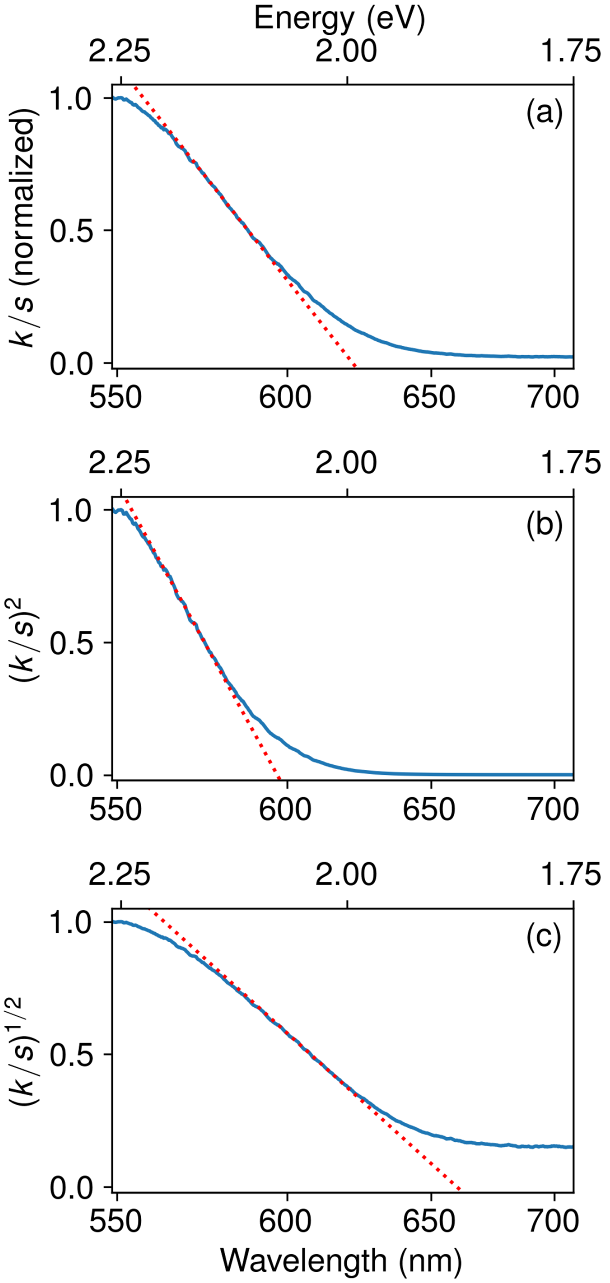

Figure S5: Diffuse reflectance spectra in the UV-visible range analyzed using different methods: (a) applying the Kubelka-Munk transformation to obtain pseudo-absorbance ($F(R) = k/s$), (b) squaring $F(R)$ to linearize direct band gaps associated with bands exhibiting parabolic dispersion, and (c) taking the square root of $F(R)$ to linearize indirect band gaps with parabolic dispersion for $(NH_3(CH_2)_7NH_3)_2Sb_2I_{10}$

Diammonium Cation Length Analysis

Structures from the Cambridge Structural Database were compiled by querying structures with $(NH_3(CH_2)_nNH_3)^{2+}$ molecules where the H atoms were explicitly included and $3 \leq n \leq 12$. Only structures with 3D coordinates were retained. The database was queried August 15, 2024.

N–N distances of 731 diammonium cations were calculated from 525 crystal structures using a graph-based molecular identification algorithm implemented with the Atomic Simulation Environment (ASE)[5] and NetworkX.[6] Crystal structures were read from CIF files using ASE's io.read, which applies space group symmetry to generate the full unit cell. An undirected molecular graph was constructed for each molecule in each structure, with atoms as nodes and covalent bonds as edges; a bond was assigned between any two atoms separated by less than 1.6 Å, evaluated under the minimum image convention (MIC) to correctly handle periodic boundary conditions. Connected components of this graph were enumerated to identify individual molecules. A diammonium cation was identified as any connected component containing exactly two nitrogen atoms and n carbon atoms, where n is the number of methylene carbons in the alkyl chain. For each identified diammonium cation, the shortest bonded path between the two terminal nitrogen atoms was determined using Dijkstra's algorithm. The end-to-end N–N distance was then calculated by summing the MIC displacement vectors along each bond in this path and taking the Euclidean norm of the result; this vector-summation approach correctly resolves molecules that span a unit cell boundary, where a direct atom-to-atom distance would be artificially shortened. Distances from symmetry-equivalent molecules within the same unit cell were rounded to three decimal places, and duplicate values were removed, yielding the unique N–N distance(s) for each structure. Calculations were parallelized across all available CPU cores using joblib.[7] An interactive python notebook with working code is provided as Supporting Information (`structure_analysis_forPub.ipynb`) with the extracted distances organized by CSD entry code provided in `NNdistances.csv`. These

are available at `https://github.com/jrneilson/DiammoniumDistances`. Versions of all software libraries used: python: 3.9.15, numpy: 1.25.2, pandas: 2.2.2, networkx 2.8.8, ase: 3.22.1, joblib: 1.2.0.

For comparison of the N-N distance to the "gap spacing" in perovskite-derived structures, the gap spacing is calculated from the appropriate d-spacing that separates octahedral centers minus the average octahedron size plus the approximate ammonium radius.

References

(1) Reid, O. G.; Moore, D. T.; Li, Z.; Zhao, D.; Yan, Y.; Zhu, K.; Rumbles, G. Quantitative analysis of time-resolved microwave conductivity data. *Journal of Physics D: Applied Physics* **2017**, *50*, 493002.

(2) Earley, J. D.; Zieleniewska, A.; Ripberger, H. H.; Shin, N. Y.; Lazorski, M. S.; Mast, Z. J.; Sayre, H. J.; McCusker, J. K.; Scholes, G. D.; Knowles, R. R.; Reid, O. G.; Rumbles, G. Ion-pair reorganization regulates reactivity in photoredox catalysts. *Nature Chemistry* **2022**, 1–8.

(3) Yuan, Z. et al. Discovery of the Zintl-phosphide $BaCd_2P_2$ as a long carrier lifetime and stable solar absorber. *Joule* **2024**, *8*, 1412–1429.

(4) Peters, A. N.; Jordano, P. A.; Taylor, J. A.; Reid, O. G.; Neilson, J. R. Shifting Defect Self-Regulation via Disordered Vacancies in Hollow Tin Perovskites. *Chemistry of Materials* **2026**,

(5) Hjorth Larsen, A. et al. The atomic simulation environment—a Python library for working with atoms. *Journal of Physics: Condensed Matter* **2017**, *29*, 273002.

(6) Hagberg, A. A.; Schult, D. A.; Swart, P. J. Exploring Network Structure, Dynamics,

and Function using NetworkX. Proceedings of the 7th Python in Science Conference. Pasadena, CA USA, 2008; pp 11 – 15.

(7) Joblib Development Team Joblib: running Python functions as pipeline jobs. 2026; `https://joblib.readthedocs.io/`.
